# He-Jie-Shen-Shi Decoction as an Adjuvant Therapy on Severe Coronavirus Disease 2019: A Retrospective Cohort and Potential Mechanistic Study

**DOI:** 10.3389/fphar.2021.700498

**Published:** 2021-06-18

**Authors:** Haibo Hu, Kun Wang, Li Wang, Yanjun Du, Juan Chen, Yongchun Li, Chuanbo Fan, Ning Li, Ying Sun, Shenghao Tu, Xuechao Lu, Zhaoshan Zhou, Huantian Cui

**Affiliations:** ^1^ Qingdao Hospital of Traditional Chinese Medicine (Qingdao Hiser Hospital), Qingdao, China; ^2^ College of Acupuncture and Orthopedics, Hubei University of Chinese Medicine, Wuhan, China; ^3^ Tongji Hospital, Tongji Medical College, Wuhan, China; ^4^ Qingdao Municipal Hospital, Qingdao, China; ^5^ Shandong Provincial Key Laboratory of Animal Cell and Developmental Biology, School of Life Sciences, Shandong University, Qingdao, China

**Keywords:** coronavirus disease 2019, acute respiratory distress syndrome, he-jie-shen-shi decoction, retrospective cohort study, network pharmacology

## Abstract

Combination therapy using Western and traditional Chinese medicines has shown notable effects on coronavirus disease 2019 (COVID-19). The He-Jie-Shen-Shi decoction (HJSS), composed of *Bupleurum chinense* DC., *Scutellaria baicalensis* Georgi, *Pinellia ternata* (Thunb.) Makino, *Glycyrrhiza uralensis* Fisch. ex DC., and nine other herbs, has been used to treat severe COVID-19 in clinical practice. The aim of this study was to compare the clinical efficacies of HJSS combination therapy and Western monotherapy against severe COVID-19 and to study the potential action mechanism of HJSS. From February 2020 to March 2020, 81 patients with severe COVID-19 in Wuhan Tongji Hospital were selected for retrospective cohort study. Network pharmacology was conducted to predict the possible mechanism of HJSS on COVID-19-related acute respiratory distress syndrome (ARDS). Targets of active components in HJSS were screened using the Traditional Chinese Medicine Systems Pharmacology (TCMSP) and PharmMapper databases. The targets of COVID-19 and ARDS were obtained from GeneCards and Online Mendelian Inheritance in Man databases. The key targets of HJSS in COVID-19 and ARDS were obtained based on the protein–protein interaction network (PPI). Kyoto Encyclopedia of Genes and Genomes analysis (KEGG) was conducted to predict the pathways related to the targets of HJSS in COVID-19 and ARDS. A “herb-ingredient-target-pathway” network was established using Cytoscape 3.2.7. Results showed that the duration of the negative conversion time of nucleic acid was shorter in patients who received HJSS combination therapy. HJSS combination therapy also relieved fever in patients with severe COVID-19. Network pharmacology analysis identified interleukin (IL) 6, tumor necrosis factor (TNF), vascular endothelial growth factor A (VEGFA), catalase (CAT), mitogen-activated protein kinase (MAPK) 1, tumor protein p53 (TP53), CC-chemokine ligand (CCL2), MAPK3, prostaglandin-endoperoxide synthase 2 (PTGS2), and IL1B as the key targets of HJSS in COVID-19-related ARDS. KEGG analysis suggested that HJSS improved COVID-19-related ARDS by regulating hypoxia-inducible factor (HIF)-1, NOD-like receptor, TNF, T cell receptor, sphingolipid, PI3K-Akt, toll-like receptor, VEGF, FoxO, and MAPK signaling pathways. In conclusion, HJSS can be used as an adjuvant therapy on severe COVID-19. The therapeutic mechanisms may be involved in inhibiting viral replication, inflammatory response, and oxidative stress and alleviating lung injury. Further studies are required to confirm its clinical efficacies and action mechanisms.

## Introduction

Since its outbreak in 2019, coronavirus disease 2019 (COVID-19) has become a global pandemic (Jin et al., 2020). Current epidemiological studies have shown more than 130 million people worldwide have been diagnosed (Boban et al., 2021). The pandemic has caused more than 2.9 million deaths worldwide, and the mortality cases continue to rise (Novelli et al., 2020). Severe acute respiratory syndrome coronavirus 2 (SARS-CoV-2), the virus that causes COVID-19, infects host cells by binding with angiotensin-converting enzyme 2 (ACE2) mainly in the respiratory system, thereby impairing it, as indicated by fever, coughing, shortness of breath, and chest tightness, among other symptoms (Farsalinos et al., 2020; Gao et al., 2020). ACE2 is also expressed on enterocytes, cardiomyocytes, renal proximal tubules, and neurocytes (Xu et al., 2020). Some patients with SARS-CoV-2 infection have also exhibited multi-system symptoms including diarrhea, acute cardiac injury, abnormal renal function, and myalgia (Adapa et al., 2020; Asadi-Pooya et al., 2020; Nishiga et al., 2020; Villapol et al., 2020). Patients with severe COVID-19 tend to develop acute respiratory distress syndrome (ARDS) and multiple organ failure (MOF), which are the major fatal events of COVD-19 (Li et al., 2020; Iwasaki et al., 2021). Clinical studies have further shown that recovered patients can develop sequelae such as interstitial lung disease and hyposmia, which can have long-term impacts on the quality of life (Mihai et al., 2020; Meng et al., 2020).

To date, no effective drugs have been developed to treat COVID-19 (Stasi et al., 2020). Preliminary clinical studies have demonstrated that remdesivir could improve lower respiratory tract infection; however, the safety and efficacy of remdesivir still need to be established (Beigel et al., 2020; Spinner et al., 2020; Wang F. et al., 2020; Wang Y. et al., 2020). Although several vaccines against SARS-CoV-2 infection have passed their phase III trials and some countries have a good vaccination rate, the uncertainty of long-term immunity after vaccination and the potential to develop severe side effects such as anaphylactic shock and thrombosis are also important considerations when opting for vaccination. (Zhu et al., 2020). Given the limitations of current therapeutics and the severity of the disease, the development of new therapies for COVID-19 are urgently needed (Ren et al., 2020; Kwok et al., 2021).

Traditional Chinese medicine (TCM) has gained abundant therapeutic knowledge from treating various diseases for thousands of years. TCM-based treatments for SARS, Middle East respiratory syndrome, and H7N9 have achieved significant therapeutic effects (Zhang et al., 2020). TCM-based treatments have achieved favorable results during the outbreak of COVID-19 early in 2020 (Cui et al., 2020). A meta-analysis indicated that combining the Lian-Hua-Qing-Wen granule and conventional treatment showed a higher overall effective rate on COVID-19 compared with the conventional treatment alone (Liu et al., 2021). “Fei Yan No. 1” could significantly improve the clinical outcomes and reduce the duration of the negative conversion time of nucleic acid in patients with COVID-19 (Ai et al., 2020). Tanreqing Capsule treatment also reduced the duration of the negative conversion time of nucleic acid and increased CD3^+^ T cell production in patients with COVID-19 (Zhang et al., 2021). The Qingfei Paidu decoction combined with Western medicine improved the blood test results, inflammatory factors, and multi-organ biochemical indices in patients with COVID-19 (Chen et al., 2020; Yang et al., 2020). A retrospective cohort study showed that individualised Chinese medicine could improve the level of D-dimer and reduce the mortality in patients with COVID-19 (Shu et al., 2021).

He-Jie-Shen-Shi decoction (HJSS) has been used for the treatment of severe COVID-19 in clinical practice. It is composed of *Bupleurum chinense* DC., *Scutellaria baicalensis* Georgi, *Pinellia ternata* (Thunb.) Makino, *Glycyrrhiza uralensis* Fisch. ex DC., *Codonopsis pilosula* (Franch.) Nannf., *Poria cocos* (Schw.) Wolf, *Alisma plantago-aquatica subsp. orientale* (Sam.) Sam., *Atractylodes macrocephala* Koidz., *Neolitsea cassia* (L.) Kosterm., *Coix lacryma-jobi var. ma-yuen* (Rom.Caill.) Stapf, *Pyrrosia lingua* (Thunb.) Farw., *Plantago asiatica* L. and *Benincasa hispida* (Thunb.) Cogn.

Here, the therapeutic effects of HJSS on patients with severe COVID-19 were evaluated in a retrospective cohort study and the possible mechanisms were predicted using network pharmacology.

## Clinical Efficacy Study

### Study Design and Participants

This study was approved by the Institutional Ethics Board of Wuhan Tongji Hospital (Approval No. TJ-C20200112). Informed consent was waived since this was a retrospective study. Consecutive patients hospitalized from February 9th to March 31st, 2020, in Wuhan Tongji Hospital were enrolled.

### Inclusion Criteria

All patients (age range, 18–75) who were diagnosed with severe COVID-19 according to the Protocol for Diagnosis and Treatment of COVID-19 guidelines (4th–6th editions) issued by the National Health Commission of China (General Office of The National Health and Health Commission, 2020) were included in the study (Supplementary Table S1).

### Exclusion Criteria

1) Pregnant or lactating women; 2) severe primary diseases such as severe combined immune deficiency disease, congenital pulmonary airway malformation, congenital heart disease, and bronchopulmonary dysplasia; 3) psychiatric or neurological disorder; 4) transfer to other hospitals; and 5) other factors such as self-withdrawal during treatment and incomplete clinical data records (Supplementary Table S1).

### Preparation of HJSS

HJSS granules were provided by the Wuhan Tongji Hospital (Tianjin, China). The components of HJSS are shown in Table 1. The herb granules were fully dissolved in 200 ml hot water (>70°C).

**TABLE 1:** Components of HJSS.

Chinese name	Latin nam	Dose (grams)
Chai Hu	*Bupleurum chinense* DC.	18
Huang Qin	*Scutellaria baicalensis* Georgi	9
Ban Xia	*Pinellia ternata* (Thunb.) Makino	9
Gan Cao	*Glycyrrhiza uralensis* Fisch.ex DC.	6
Dang Shen	*Codonopsis pilosula* (Franch.) Nannf.	9
Fu Ling	*Poria cocos* (Schw.)Wolf	18
Ze Xie	*Alisma plantago-aquatica subsp. orientale* (Sam.) Sam.	12
Bai Zhu	*Atractylodes macrocephala* Koidz.	12
Gui Zhi	*Neolitsea cassia* (L.) Kosterm.	6
Yi Yi Ren	*Coix lacryma-jobi var. ma-yuen* (Rom.Caill.) Stapf	18
Shi Wei	*Pyrrosia lingua* (Thunb.) Farw.	12
Che Qian Zi	*Plantago asiatica* L.	12
Dong Gua Ren	*Benincasa hispida* (Thunb.) Cogn.	15

## Quality Control of HJSS

### Sample Preparation

12 principal components in HJSS, including atractylenolide III, saikosaponin A, saikosaponin D, lobetyolin, baicalin, pachymic acid, liquiritin, L-arginine, chlorogenic acid, geniposidic acid, cinnamic acid and alisol B-23-acetate were used as the reference standards according to the quality control methods in Pharmacopoeia of People’s Republic of China (2020 edition) issued by Chinese Pharmacopoeia Commission. Briefly, 5 mg of each reference standard was dessolved in 5 ml methanol and incubated at 50°C for 30 min to obtain the stock solution of each reference standard (1 mg/ml). Then, 100 μL of stock solution was dessolved in 900 μL methanol to obtain the test solutions of reference standards. Besides, 300 μL of HJSS were fully dessolved in 900 μL methanol and centrifuged at 13,000 rpm for 10 min and the supernatant was collected to obtain the test solution of HJSS.

### Ultra Performance Liquid Chromatography—Mass Spectrometer

Ultra performance liquid chromatography (UPLC; ACQUITY UPLC®, United States) coupled with Xevo G2 quadrupole-time-of-flight (Q-TOF) mass spectrometer (MS; Waters Corp., Milford, MA, United States) systems were used as the quality control of HJSS. 2 μL of each test solution was injected onto an ACQUITY UPLC BEH C18 Column (2.1 × 100 mm, 1.7 μm; column temperature: 50°C; flow rate: 0.3 ml/min). Mobile phase A was 0.1% formic acid aqueous solution and mobile phase B was acetonitrile contained 0.1% formic acid. The mobile phase conditions were: 0 min, 5% B; 1 min, 10% B; 6 min 60% B; 6.5 min 100% B; 10 min 100% B; 10.1 min 5% B; 13 min 5% B.

A Q-TOF MS equipped with an electrospray ionization (ESI) source was used for both positive and negative ionization scan modes (50–1,200 Da). The scan time was 0.2 s.

The detailed parameters of MS were: capillary voltage of 3,000 V (positive mode) and 2,200 V (negative mode), desolvation temperature at 350°C, sample cone voltage of 40 V, extraction cone voltage of 4 V, source temperature of 100°C, cone gas flow of 40 L/h and desolvation gas flow of 800 L/h (both positive and negative modes).

### Clinical Treatments and Data Collection

Of the 109 patients assessed for eligibility, 28 were excluded, including one pregnant woman, seven cases with severe primary diseases, four cases with psychiatric or neurological disorder, seven cases that had transferred to other hospitals, and nine cases with incomplete clinical data records. There were no significant differences in baseline characteristics between 28 excluded patients and 81 included patients. A final total of 81 patients were included and divided into the general treatment (GT) and HJSS + GT groups. Baseline data were collected.

The GT group received GT including oxygen inhalation therapy (high-flow nasal cannula oxygen therapy and mechanical ventilation if necessary), parenteral nutrition, antiviral therapy (arbidol, 200 mg, per os three times daily) and other treatments based on the Protocol for Diagnosis and Treatment of COVID-19 guidelines (4th–6th editions). The HJSS + GT group received GT and HJSS (per os twice daily).

**FIGURE 1:**
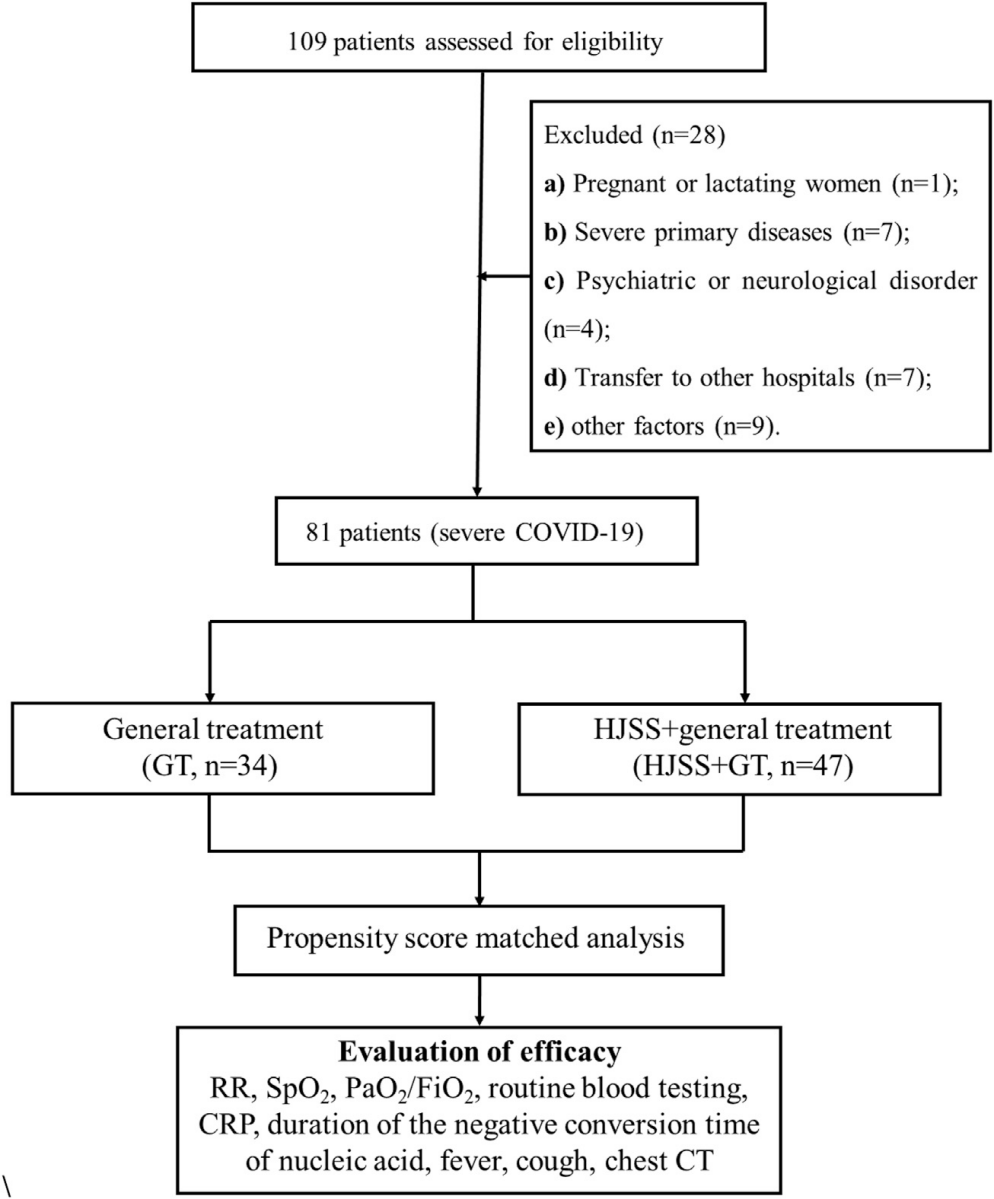
Data collection of clinical efficacy study.

Clinical characteristics, respiratory rate (RR), blood oxygen saturation (SpO_2_), ratio of arterial partial pressure of oxygen to fraction of inspired oxygen (PaO_2_/FiO_2_), blood laboratory tests, and chest computed tomography (CT) images taken during hospitalization were collected to observe the effects of HJSS and GT on severe COVID-19. Clinical characteristics included the duration of the negative conversion time of nucleic acid and the duration of fever and cough. Blood laboratory tests included routine blood tests and C-reactive protein (CRP) (Figure 1).

### Statistical Analysis

Propensity score matching (PSM) was used to compensate for differences in baseline characteristics between GT and HJSS + GT groups. Propensity scores were calculated by logistic regression using demographic and clinical characteristics of the study population (age, sex, diabetes, hypertension, coronary artery disease, and chronic kidney disease). A caliper of width equal to 0.02 of the standard deviation of the logit of the propensity score was applied. To better match GT and HJSS + GT groups, we used the 1:1 ratio matching method. Covariate balance before and after propensity score matching was assessed using standardized differences. A standardized difference of <0.1 was considered negligible, and balance was assumed to be met.

The Shapiro-Wilks test was used to test for normality. For continuous variables that conform to a normal distribution, we used the mean and standard deviation to describe the data distribution, and two-sample *t* test was used to compare the differences between GT and HJSS + GT groups. For continuous variables that do not satisfy a normal distribution, we used median and interquartile range to describe the data distribution, and Wilcoxon rank-sum test was used to compare the differences between GT and HJSS + GT groups. For categorical variables, frequency and percentage were used to describe the data distribution, and the chi-square test or Fisher’s exact test were used to compare the differences between GT and HJSS + GT groups. Considering that the baseline values of dependent variables may have some impact on the comparison between the groups, we defined the differences of dependent variables, and intergroup comparisons for the differences of dependent variables were analyzed using the two-sample *t* test or Wilcoxon rank-sum test.

For all analysis, we used SPSS version 20.0 (SPSS, Inc., Chicago, IL, United States). A *p*-value < 0.05 was considered statistically significant. Curve fitting was carried out using the GraphPad Prism five graphical package (GraphPad Software, Inc., La Jolla, CA, United States).

## Network Pharmacology

### Virtual Screening for Active Ingredients of HJSS

A search of the Traditional Chinese Medicine Systems Pharmacology Database and Analysis Platform (TCMSP: https://tcmspw.com/tcmsp.php) was performed to generate candidate ingredients of HJSS using the following keywords: “*Bupleurum chinense* DC,.” “*Scutellaria baicalensis* Georgi,” “*Pinellia ternata* (Thunb.) Makino,” “*Glycyrrhiza uralensis* Fisch. ex DC.,” “*Codonopsis pilosula* (Franch.) Nannf.,”“*Poria cocos* (Schw.) Wolf,” “*Alisma plantago-aquatica subsp. orientale* (Sam.) Sam.,”“*Atractylodes macrocephala* Koidz.,” “*Neolitsea cassia* (L.) Kosterm.” “*Coix lacryma-jobi var. ma-yuen* (Rom.Caill.) Stapf,” “*Pyrrosia lingua* (Thunb.) Farw.,” “*Plantago asiatica* L.” and “*Benincasa hispida* (Thunb.) Cogn.” To obtain the active ingredients of HJSS, these ingredients were further screened for oral bioavailability (OB ≥ 30%) and drug-like criteria (DL ≥ 0.18).

### Potential Target Prediction and Gene Name Annotation

The chemical ingredients of HJSS obtained from TCMSP were input into the PubChem database (https://pubchem.ncbi.nlm.nih.gov) for 3D structure identification. The 3D structures of the ingredients were then uploaded to the PharmMapper platform server (http://www.lilab-ecust.cn/pharmmapper/) to generate relevant targets for the active ingredients. The UniProt database (https://www.uniprot.org/) was then used to standardize the target proteins per the active ingredients and obtain the gene names per the targets.

### Targets Screening of ARDS and COVID-19

The Online Mendelian Inheritance in Man (OMIM) (http://omim.org) and GeneCards (https://www.genecards.org) databases were searched for relevant targets using the keyword “Acute Respiratory Distress Syndrome (ARDS)” and “COVID-19”. The Venny online interactive platform version 2.1 (http://bioinfogp.cnb.csic.es/tools/venny/) was used to intersect the targets of HJSS, ARDS, and COVID-19 to further isolate treatment targets and obtain the potential targets of HJSS in COVID-19-related ARDS (Yang et al., 2020).

### Protein–Protein Interaction Network

The protein–protein interaction (PPI) of potential targets of HJSS in COVID-19-related ARDS were studied *via* the STRING database. In addition, Cytoscape 3.7.2 (http://www.cytoscape.org/) was used to construct the PPI network. The interactions between the proteins in the PPI network were analyzed using the MCODE plugin on Cytoscape 3.7.2. Proteins with many interactions showed larger numbers and widths of connections. The top ten highly interactive proteins were then identified as the key targets (Yang et al., 2020).

### Pathway Analysis and Construction of the “Herb-Ingredient-Target-Pathway” Network

The intersecting targets of HJSS, ARDS, and COVID-19 were imported into the KOBAS 3.0 database (http://kobas.cbi.pku.edu.cn) for Kyoto Encyclopedia of Genes and Genomes (KEGG) pathway analysis. The enriched KEGG terms were identified and analyzed based on *p*-value correction <0.05 (Bonferroni step down). Cytoscape 3.7.2 was then used to establish the “herb-ingredient-target-pathway” network.

## Results

### Quality Control of HJSS by UPLC-MS Analysis

Atractylenolide III, saikosaponin A, saikosaponin D, lobetyolin, baicalin, pachymic acid, liquiritin, L-arginine, chlorogenic acid, geniposidic acid, cinnamic acid and alisol B-23-acetate were identified as the main components in HJSS. The detailed information of these compounds were shown in Figure S1. The typical based peak intensity (BPI) chromatograms and the characteristic fragment ions of these compounds were shown in Supplementary Figure S2 and Table S2 respectively (Supplementary meterial).

### HJSS Treatment Improved Clinical Outcomes in Severe COVID-19 Cases

**FIGURE 2:**
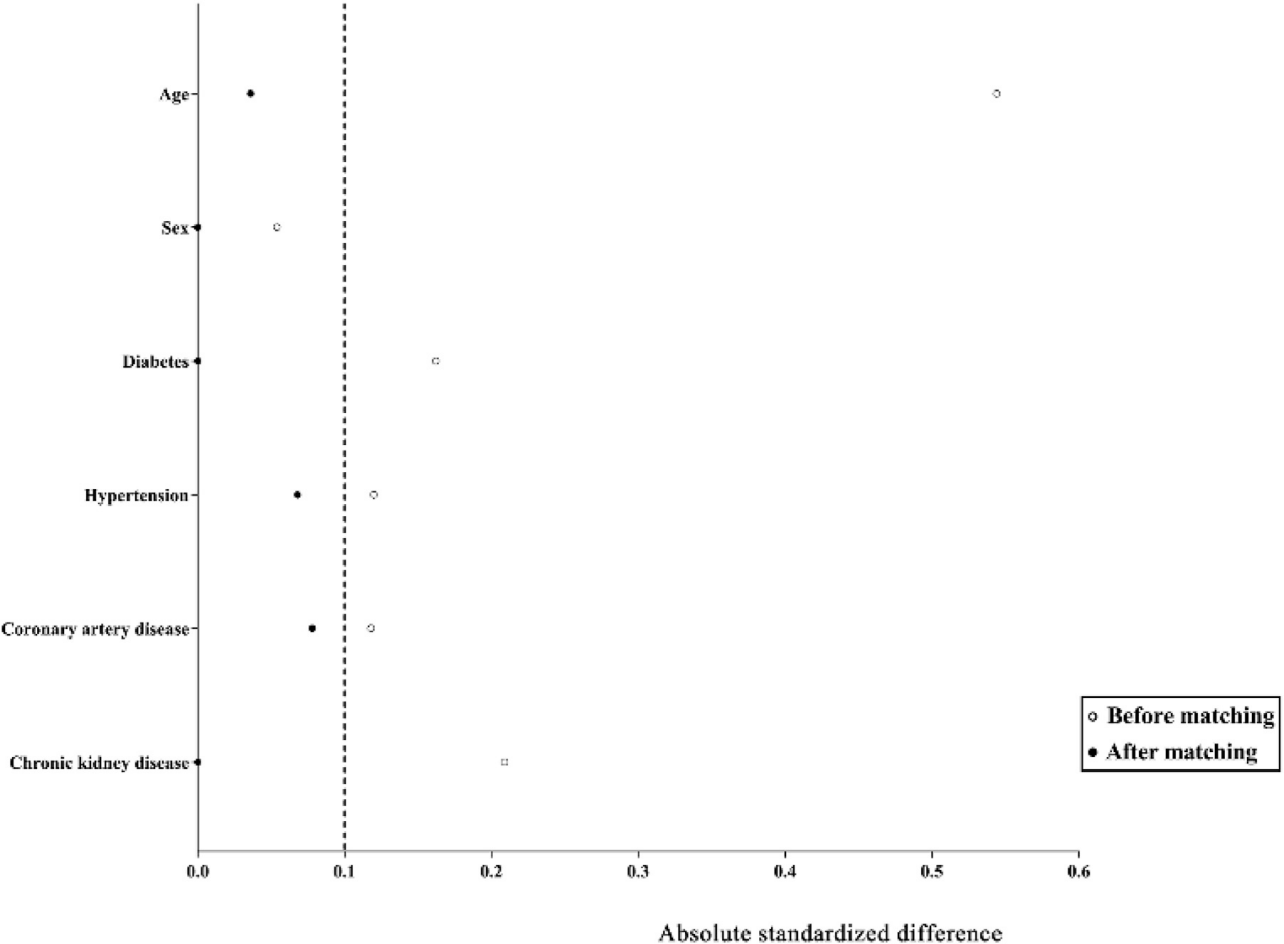
Absolute standardized differences comparing characteristics of patients in GT and HJSS + GT groups before matching and after 1:1 propensity score matching. The absolute standardized differences <0.1 show adequate matching. *Y* axis represented the selected characteristics. *X* axis of the scatterplot represented whether the status was before matching or after matching.

**FIGURE 3:**
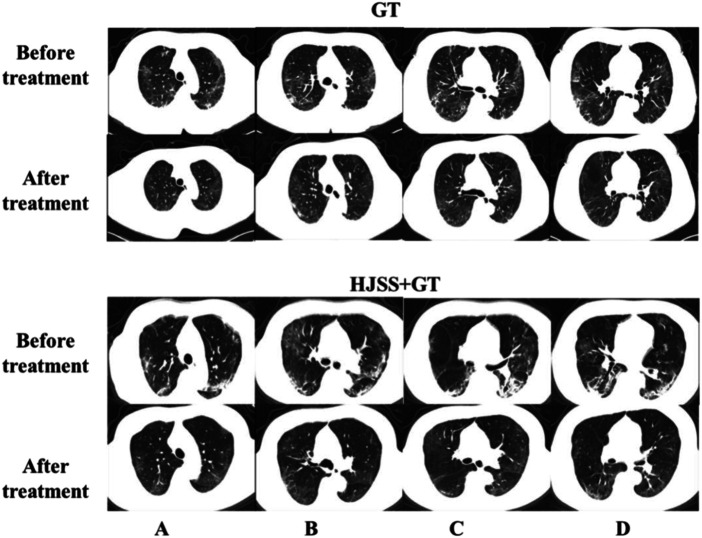
Chest CT scan before and after GT and HJSS treatment. Bilateral pulmonary infiltrate was clearly absorbed after GT and HJSS treatment. **(A–D)** represent different slices. GT group: A 66-year-old female received GT for 15 days. HJSS + GT group: A 59-year-old female received HJSS and GT for 12 days.

As shown in Table 2, no significant differences were observed in the baseline characteristics between the GT and HJSS + GT groups. After HJSS and GT treatment, the differences of clinical indicators between GT and HJSS + GT groups were shown in Table 3. For full study population, patients in HJSS + GT group were found to have a significantly lower leukocyte count compared with patients in GT group (*p* = 0.03). In addition, patients in HJSS + GT group showed a shorter duration of the negative conversion time of nucleic acid (23.09 days *vs.* 20.13 days, *p* = 0.03) and fever (15.79 days *vs.* 12.36 days, *p* = 0.03) compared with patients in GT group. We used the 1:1 PSM to generate a more balanced subsample of 32 patients in GT and HJSS + GT groups respectively. As shown in Figure 2, PSM eliminated the differences in covariate distributions, and all the standardized differences after matching were less than 0.1. For propensity score-matched subsample, we observed the consistent results for leukocyte count (*p* = 0.01), duration of the negative conversion time of nucleic acid (23.34 days *vs.* 21.03 days, *p* = 0.04) and fever (15.75 days *vs.* 12.53 days, *p* = 0.02) differences. Chest CT showed multiple ground-glass opacities and consolidation before HJSS and GT treatment. These chest CT features were significantly improved after both HJSS + GT and GT treatment (Figure 3).

**TABLE 2:** Baseline characteristic of patients in GT and HJSS + GT groups.

	References range	Full population		*p*	Propensity score-matched subsample		*p*
		GT (*n* = 34)	HJSS + GT (*n* = 47)		GT (*n* = 32)	HJSS + GT (*n* = 32)	
Age		66.5 (57–71)	63 (50–68)	0.06	66.5 (57.0–71.0)	64.5 (60.3–68.8)	0.62
Male		15 (44.12%)	22 (46.81%)	0.81	14 (43.75%)	14 (43.75%)	1.00
Fever (>37.2°C)		32 (94.12%)	44 (93.62%)	1.00	30 (93.75%)	30 (93.75%)	1.00
Cough		33 (97.06%)	45 (95.74%)	1.00	31 (96.88%)	30 (93.75%)	1.00
Underlying diseases							
Diabetes		8 (23.53%)	8 (17.02)	0.47	7 (21.88%)	7 (21.88%)	1.00
Hypertension		9 (26.47%)	15 (31.91%)	0.60	9 (28.13%)	10 (31.25%)	0.78
Coronary artery disease		7 (20.59%)	12 (25.53%)	0.60	7 (21.88%)	6 (18.75%)	0.76
Chronic kidney disease		0 (0.00%)	1 (2.13%)	1.00	0 (0.00%)	0 (0.00%)	-
Clinical indicators							
RR/min	16–20	35 (34–36)	35 (34–36)	0.50	35 (34–36)	35 (34–36)	0.84
SpO_2_ (%)	>93	85.15 ± 2.05	84.85 ± 2.27	0.55	85.25 ± 2.06	85.19 ± 2.12	0.91
PaO_2_/FiO_2_ (mmHg)	400–500	256.85 ± 16.42	253.11 ± 13.36	0.26	258.09 ± 16.11	255.16 ± 13.53	0.43
Lymphocyte count (×10^9^/L)	1.1–3.2	0.89 ± 0.20	0.96 ± 0.21	0.11	0.88 ± 0.20	0.98 ± 0.24	0.07
Leukocyte count (×10^9^/L)	3.5–9.5	4.93 (3.52–6.38)	5.78 (4.57–7.47)	0.13	4.85 (3.48–6.29)	5.96 (5.06–7.74)	0.05
Neutrophil count (×10^9^/L)	1.8–6.3	3.51 (2.28–5.68)	3.98 (2.62–6.19)	0.26	3.51 (2.31–5.74)	4.13 (2.78–7.22)	0.16
CRP (mg/L)	<10	27.05 (22.80–31.80)	25.00 (18.80–32.50)	0.38	27.39 ± 6.72	25.14 ± 7.99	0.23

**TABLE 3:** Comparison of treatment responses between GT and HJSS + GT groups.

	Full population		*p*	Propensity score-matched subsample		*p*
	GT (*n* = 34)	HJSS + GT (*n* = 47)		GT (*n* = 32)	HJSS + GT (*n* = 32)	
RR difference/min	−10.53 ± 2.81	−11.19 ± 2.42	0.26	−10.59 ± 2.65	−10.94 ± 2.55	0.60
SpO_2_ difference (%)	10.41 ± 2.13	11.00 ± 2.43	0.26	10.34 ± 2.15	10.91 ± 2.40	0.33
PaO_2_/FiO_2_ difference (mmHg)	100.50 (83.00–117.25)	103.00 (95.00–−115.00)	0.64	100.59 ± 21.59	100.81 ± 18.20	0.97
Lymphocyte count difference (×10^9^/L)	0.44 ± 0.25	0.50 ± 0.28	0.32	0.43 ± 0.24	0.50 ± 0.29	0.27
Leukocyte count difference (×10^9^/L)	1.31 ± 2.98	−0.13 ± 2.94	0.03	1.38 ± 2.99	−0.57 ± 2.55	0.01
Neutrophil count difference (×10^9^/L)	−0.05 (−1.76–1.45)	−0.41 (−1.63–0.28)	0.26	−0.23 (−1.83–1.41)	−0.84 (−2.15–0.23)	0.22
CRP difference (mg/L)	−17.49 ± 6.39	−17.58 ± 5.74	0.95	−17.62 ± 6.54	−17.44 ± 5.92	0.91
Nucleic acid RT-PCR negative (days)	23.09 ± 6.38	20.13 ± 5.37	0.03	23.34 ± 6.25	21.03 ± 5.42	0.04
Fever (days)	15.79 ± 7.31	12.36 ± 6.36	0.03	15.75 ± 7.51	12.53 ± 6.70	0.02
Cough (days)	21.06 ± 8.63	20.11 ± 9.98	0.66	20.84 ± 8.82	18.06 ± 9.41	0.23

### Screening the Active Ingredients of HJSS

**FIGURE 4:**
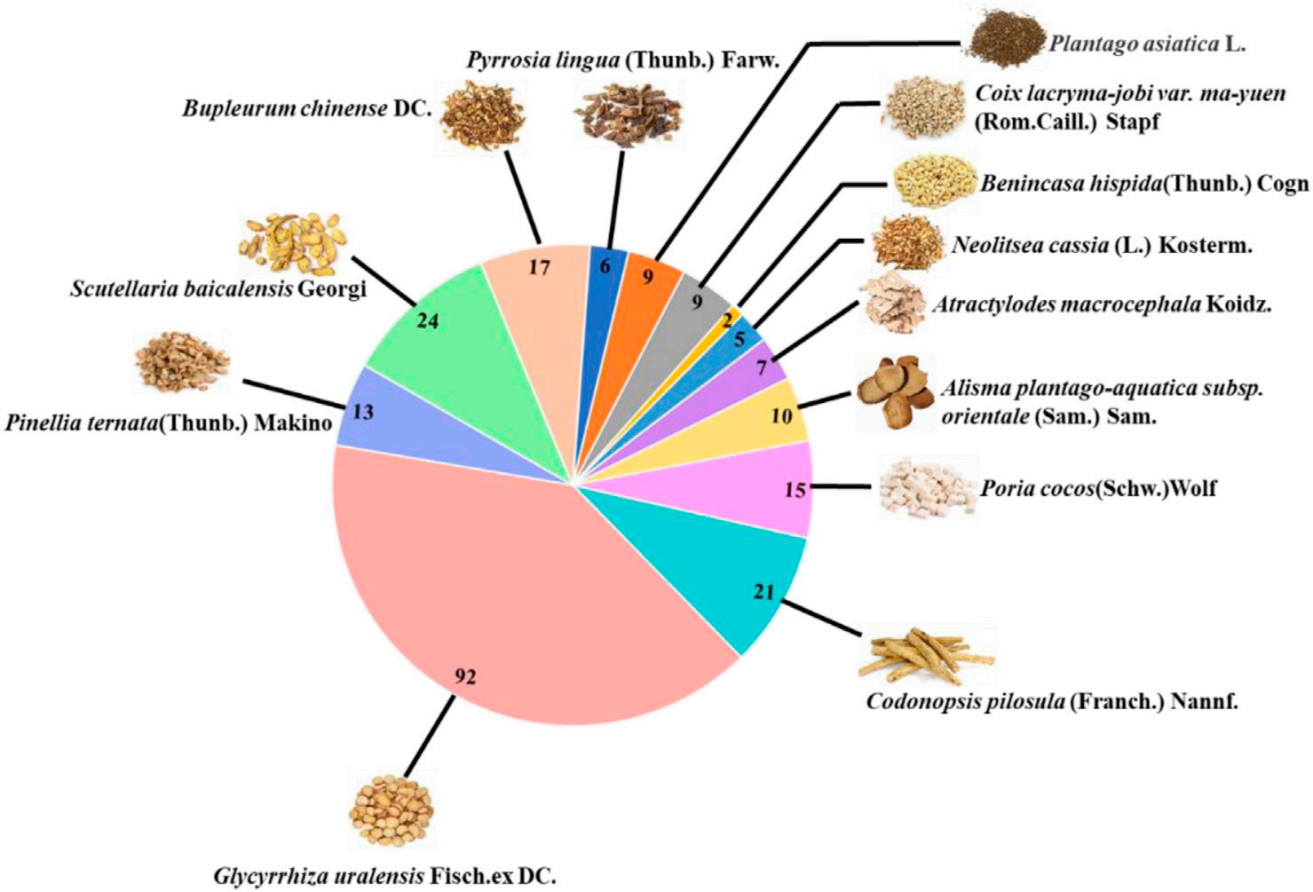
The number of active ingredients of each herb in HJSS obtained from TCMSP (OB ≥ 30%, DL ≥ 0.18).

A total of 208 active ingredients were obtained from the screening (OB ≥ 30% and DL ≥ 0.18), including Shiwei [Pyrrosia lingua (Thunb.) Farw.] 6, Cheqianzi (*Plantago asiatica* L.) 9, Yiyiren (*Coix lacryma-jobi var. ma-yuen* (Rom.Caill.) Stapf) 9, Dongguaren [*Benincasa hispida* (Thunb.) Cogn.] 2, Guizhi (*Neolitsea cassia* (L.) Kosterm.) 5, Baizhu (*Atractylodes macrocephala* Koidz.) 7, Zexie [*Alisma plantago-aquatica subsp. orientale* (Sam.) Sam.] 10, Fuling (*Poria cocos* (Schw.)Wolf) 15, Dangshen [*Codonopsis pilosula* (Franch.) Nannf.] 21, Gancao (*Glycyrrhiza uralensis* Fisch. ex DC.) 92, Banxia (*Pinellia ternata* (Thunb.) Makino) 13, Huangqin (*Scutellaria baicalensis* Georgi) 24, and Chaihu (*Bupleurum chinense* DC.) 17 (Figure 4). The detailed information of active ingredients was shown in Supplementary Table S3 (Supplementary meterial).

### Identification of the Key Targets of HJSS in COVID-19-Related ARDS

**FIGURE 5:**
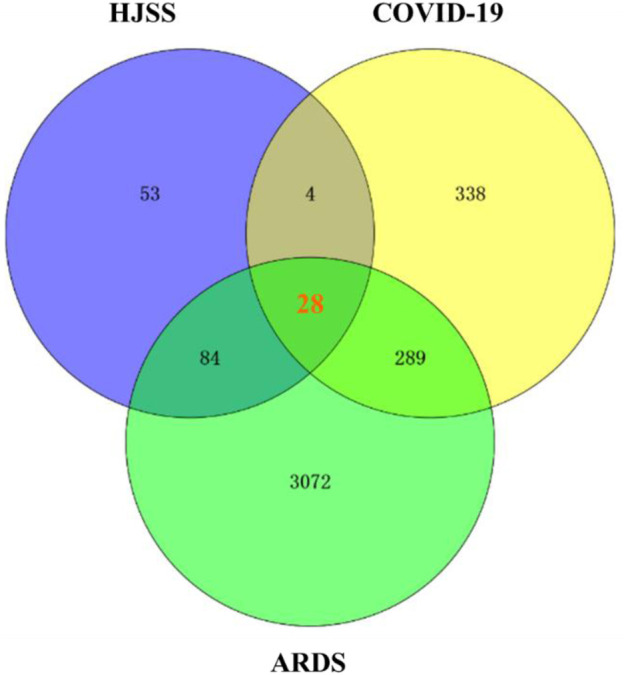
After intersecting the targets of HJSS, COVID-19 and ARDS, 28 potential targets of HJSS on COVID-19-related ARDS were obtained (Venn diagram).

**FIGURE 6:**
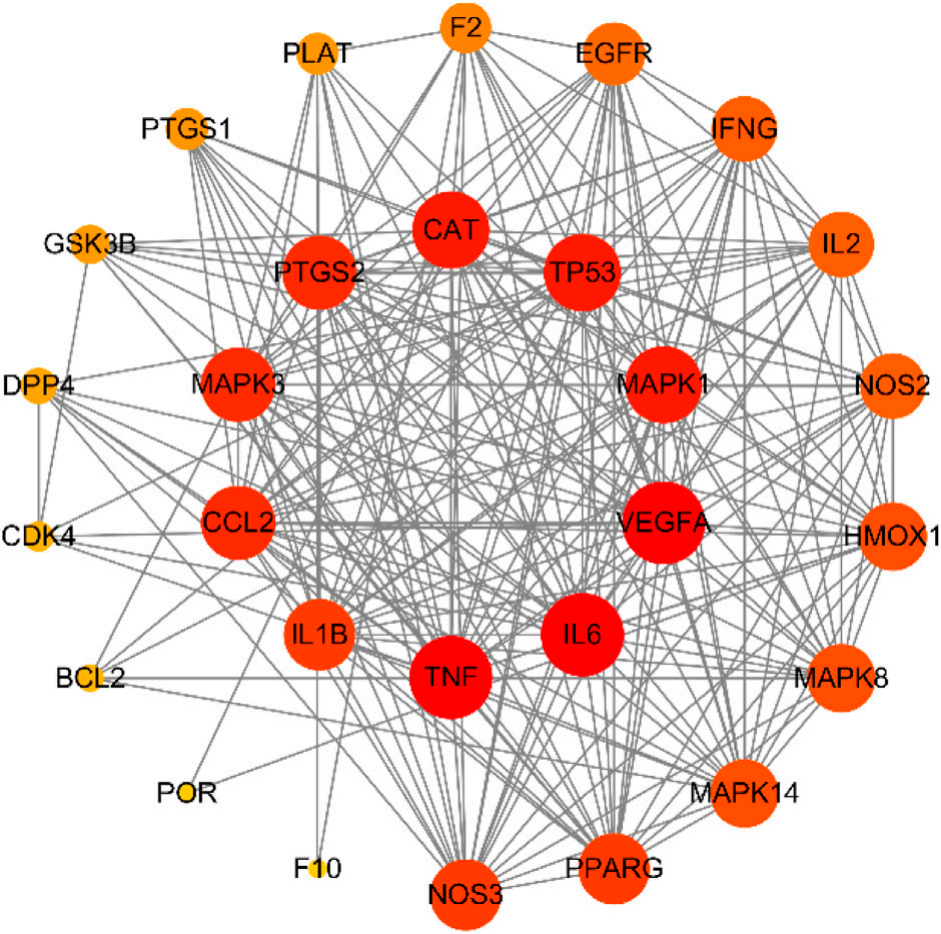
PPI network of 28 potential targets obtained from venn diagram. Nodes in red indicate more interactions with other targets, while nodes in yellow indicate less interactions with other targets.

We retrieved 169 targets from the PharmMapper platform server, based on these 208 active ingredients. Meanwhile, we obtained 659 COVID-19 targets and 3473 ARDS targets from the OMIM and GeneCards databases. By intersecting the targets of the active herb ingredients with the disease targets, we identified 28 potential targets for HJSS in COVID-19-related ARDS (Figure 5). The PPI of the 28 potential targets of HJSS in COVID-19-related ARDS were analyzed using the STRING database, and the PPI network was constructed using Cytoscape 3.7.2. The PPI network showed 181 interaction edges obtained with the 28 targets. The top 10 targets with the highest interactions were interleukin (IL) 6, tumor necrosis factor (TNF), vascular endothelial growth factor A (VEGFA), catalase (CAT), mitogen-activated protein kinase (MAPK) 1, tumor protein p53 (TP53), CC-chemokine ligand (CCL2), MAPK3, prostaglandin-endoperoxide synthase 2 (PTGS2), and IL1B. Thus, these proteins were identified as the key targets of HJSS in COVID-19-related ARDS (Figure 6).

### Development of the “Herb-Ingredient-Target-Pathway” Network

**FIGURE 7:**
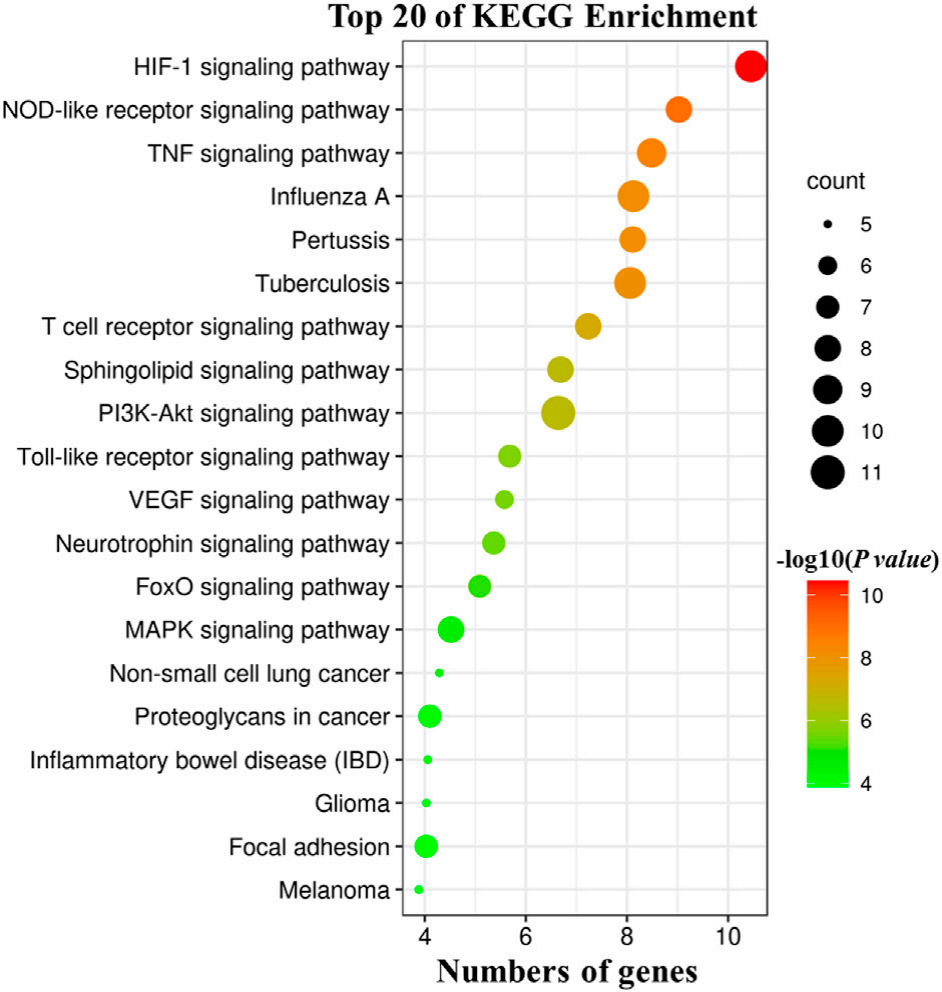
KEGG pathway analysis of 28 potential targets. The size of bubbles indicates the numbers of associated genes, the larger bubble indicates more gene counts. Color coding scale indicates the significance of KEGG terms, the deeper red indicates lower *p* values [higher -log10 (*p* value)].

**FIGURE 8:**
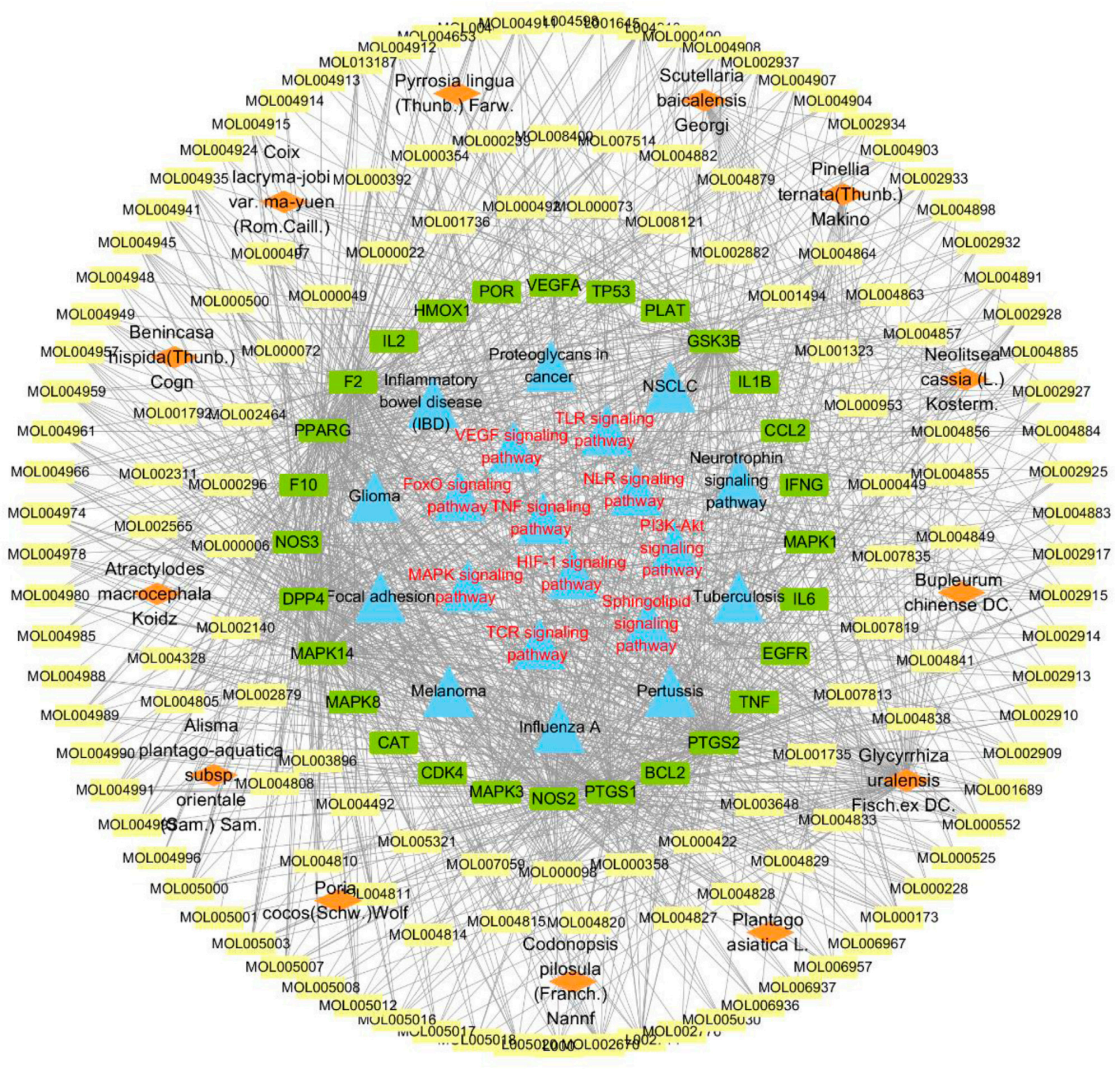
“Herb-ingredient-target-pathway” network of HJSS on COVID-19-related ARDS.

According to the KEGG pathway analysis of 28 potential targets, 181 KEGG terms were enriched and the top twenty pathways with highest significance (lowest *p* value) were showed in Figure 7. Potential mechanistic pathways of HJSS in COVID-19-related ARDS were primarily related to the HIF-1, NOD-like receptor, TNF, T cell receptor, sphingolipid, PI3K-Akt, toll-like receptor, VEGF, FoxO, and MAPK signaling pathways. Furthermore, the herbs, ingredients, targets, and pathways of the HJSS therapeutic mechanism in COVID-19-related ARDS were imported into Cytoscape 3.7.2 to generate the “herb-ingredient-target-pathway” network. In the “herb-ingredient-target-pathway” network diagram, the orange rhombic nodes represent herbs, the yellow rectangle nodes represent active ingredients, the green rectangle nodes represent targets, and the blue rectangle nodes represent pathways (Figure 8).

## Discussion

In this study, we compared the clinical efficacies of HJSS + GT and GT on severe COVID-19. Our results showed that HJSS + GT shortened the duration of the negative conversion time of nucleic acid and relieved fever. The negative conversion time of nucleic acid is the most direct diagnostic indicator of recovery from COVID-19. Fever is a primary clinical outcome in severe COVID-19 (Bolay et al., 2020). Accelerating the treatment of fever consequently lowers inflammatory levels. Routine blood testing is an easy and efficient way to understand the severity of COVID-19 (Ferrari et al., 2020; Lu et al., 2021). Our results showed lower total lymphocyte count and higher level of CRP in patients with severe COVID-19. In COVID-19, the total lymphocyte count is decreased because during infection, SARS-CoV-2 enters the lymph nodes, spleen, and other immune system organs to replicate and destroys the lymphocytes thereafter (Kim et al., 2020). CRP is also an important laboratory test index reflecting the level of inflammatory response (Felger et al., 2020). Our results also showed lower leukocyte count in HJSS + GT group compared with GT group. However, the difference in leukocyte count may be cause by the individual differences of patients in each group according to the reference range. Furthermore, most patients also exhibited higher RR, lower SpO_2_ and PaO_2_/FiO_2_ and cough, chest CT scans revealed multiple ground-glass opacities and consolidation in severe COVID-19, which fulfill the diagnostic criteria for severe COVID-19 (Li et al., 2020; Pan et al., 2020).

In addition, we screened the potential targets of HJSS on COVID-19 and ARDS using network pharmacology. Most severe patients with COVID-19 develop ARDS, which is a major event that can lead to death in COVID-19 (Torres et al., 2020). The key targets of HJSS identified were IL6, TNF, VEGFA, CAT, MAPK1, TP53, CCL2, MAPK3, PTGS2, IL1B—chemokines and pro-inflammatory highly involved in the progression of COVID-19. CCL2 could be released by multiple organs and could contribute to the inflammatory process during viral infection (Kempuraj et al., 2020). CCL2 could be released from the lungs after SARS-CoV-2 infection and could induce the activation and migration of macrophages to the lungs, initiating an inflammatory response in the lungs (Fuentes et al., 2019). The overproduction of these cytokines and chemokines could trigger hypoxia and lung injury through the impairment of the pulmonary alveoli, further decreasing the capacity of the airway lumen (Ci et al., 2012). Moreover, excessive pro-inflammatory cytokines could lead to an inflammatory cytokine storm, triggering MOF (Wang H. et al., 2020; Wang J. et al., 2020; Karki et al., 2021). MAPK could be activated by pro-inflammatory cytokines to subsequently upregulate the gene expression of pro-inflammatory cytokines and contribute to the progression of the inflammatory response (Li et al., 2015). The effects of HJSS on reducing the duration of fever may in turn decrease chemokine and pro-inflammatory cytokine levels and inhibit MAPK activation. P53 is closely related to the modulation of the cell cycle, DNA repair, cell differentiation, and apoptosis (Engeland et al., 2017; Hafner et al., 2019). Studies have also shown that SARS-CoV-2 could induce the apoptosis of lymphocytes by activating the P53 signaling pathway (Xiong et al., 2020; Bordoni et al., 2021). VEGFA belongs to the PDGF/VEGF growth factor family, which participates in the progression of endothelial cell proliferation and cell migration and is necessary for angiogenesis under physiological and pathological conditions (Zhang et al., 2019). Compared with milder COVID-19 cases, the serum levels of VEGF were significantly higher in severe COVID-19 cases. The increase in VEGF levels could promote vascular permeability and contribute to lung injury (Zhang et al., 2020). Studies showed that the overproduction of reactive oxygen species (ROS) could be observed in ARDS, triggering oxidative stress and exacerbating lung injury (Gong et al., 2020). CAT is an antioxidative enzyme involved in the elimination of ROS. Since CAT activity is decreased in ARDS, promoting its expression could alleviate lung injury (Lei et al., 2021).

KEGG analysis also showed that the HIF-1, NOD-like receptor, sphingolipid, PI3K-Akt, FoxO, and T cell receptor signaling pathways may be related to the mechanisms of HJSS on COVID-19-related ARDS. HIF-1 is an important transcriptional factor induced under anoxia (Simko et al., 2017). PI3K catalyzes the transformation of PIP2 to PIP3, further triggering the phosphorylation and activation of Akt (Delaloge et al., 2017). The activation of the HIF-1 and PI3K-Akt pathways could induce the inflammatory response and angiogenesis (Palazon et al., 2014; Hou et al., 2016). NOD-like receptor, FoxO, and T cell receptor are important modulators of the inflammatory response (He et al., 2014; Hammami et al., 2018; Liu et al., 2019). As an important component of biological membranes, sphingolipids participate in various kinds of biological processes such as cell survival, differentiation, apoptosis, and migration (Hwang et al., 2017). The dysfunction in the sphingolipid pathway could disrupt the balance between cell survival and apoptosis (Hannun and Obeid, 2018). Thus, modulating the sphingolipid signaling pathway could alleviate acute lung injury (Lin et al., 2011). Based on the chest CT and network pharmacology studies, the protective effect of HJSS in lung injury may be involved in the regulation of CAT activity and the sphingolipid signaling pathway.

## Strengths and Limitations

This is a retrospective cohort and potential mechanistic study evaluating the efficacy and potential mechanism of HJSS on severe COVID-19 patients. Our data indicated that HJSS could be used as an adjuvant therapy on severe COVID-19. Compared with GT, HJSS + GT shortened the duration of the negative conversion time of nucleic acid. A shorter negative conversion time of nucleic acid could relieve pressure on the healthcare system, which is beneficial during this pandemic. Besides, we performed propensity score matched analysis to ascertain the robustness of the result. In network pharmacology study, we screened the potential targets of HJSS on severe COVID-19 by intersecting the targets of the active herb ingredients in HJSS with the targets of COVID-19 and ARDS.

Some limitations in this study should also be realized. Due to the small sample size of this retrospective study, further prospective, multi-center, double-blind and randomized controlled trials are required to evaluate the effect of HJSS in a larger patient population. The therapeutic mechanisms of HJSS still need to be verified. Virus-induced ARDS animal models could be used in further studies to validate the effects and mechanisms of HJSS on severe COVID-19. Component analyses such as high-performance liquid chromatography could also reveal the main components of HJSS. The main components could then be purified and studied in animal (virus-induced ARDS) and cell models (pseudoviral infection and virus-induced inflammation).

## Conclusion

In conclusion, HJSS can be used as an adjuvant therapy on severe COVID-19. The therapeutic mechanisms may be involved in inhibiting viral replication, inflammatory response, and oxidative stress and alleviating lung injury. Further studies are required to confirm its clinical efficacies and action mechanisms.

## Data Availability

The raw data supporting the conclusions of this article will be made available by the authors, without undue reservation.
