# Supplementary material for: He-Jie-Shen-Shi Decoction as an Adjuvant Therapy on Severe Coronavirus Disease 2019: A Retrospective Cohort and Potential Mechanistic Study
Source: Front Pharmacol. 2021 Jun 18;12:700498. doi: 10.3389/fphar.2021.700498 (PMC8250425; doi:10.3389/fphar.2021.700498)
Supplement: Supplementary file 1 [file DataSheet1.docx]

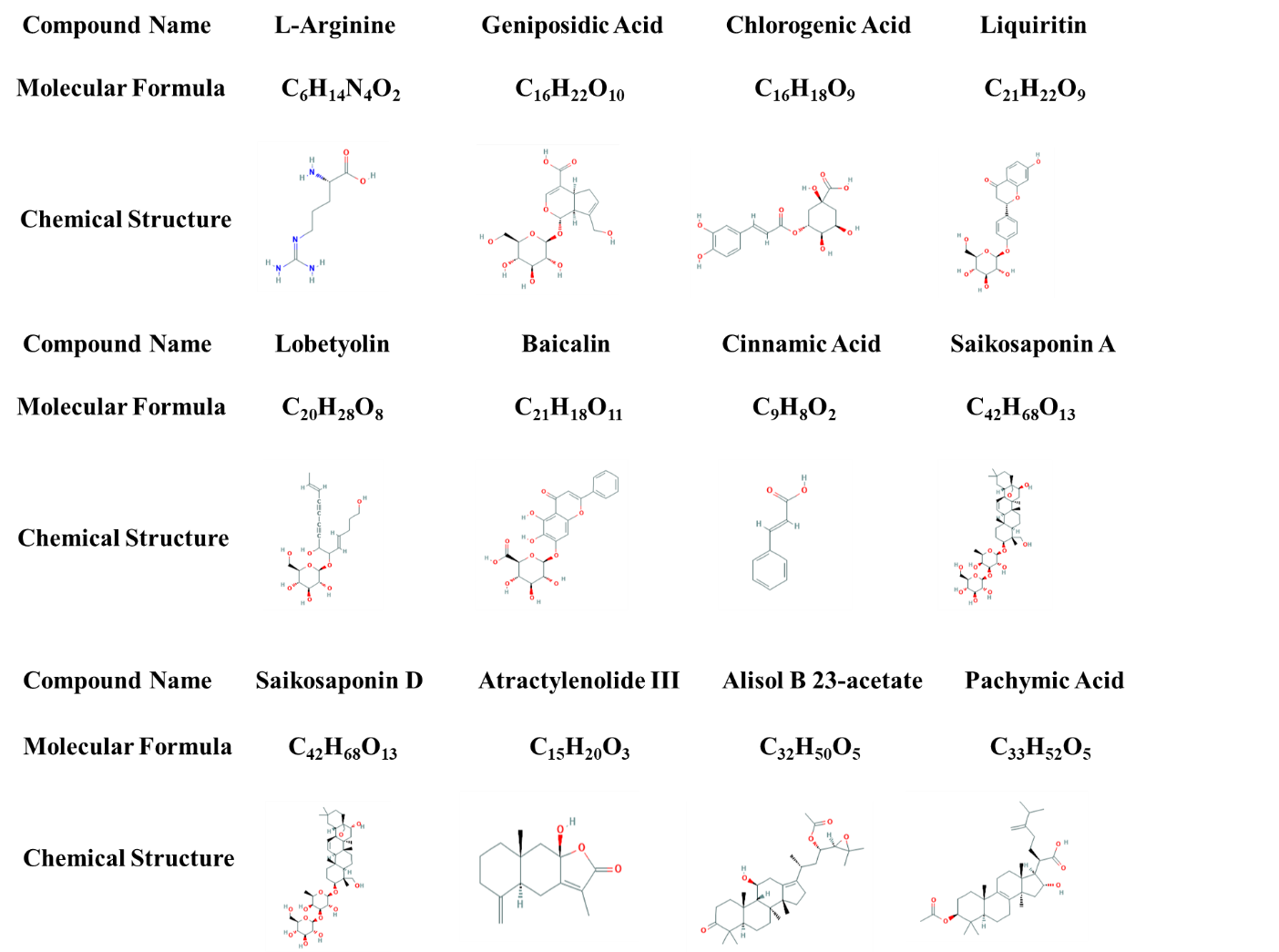


**FIGURE S1:** The molecular formulas and chemical structures of reference standards.

**a**


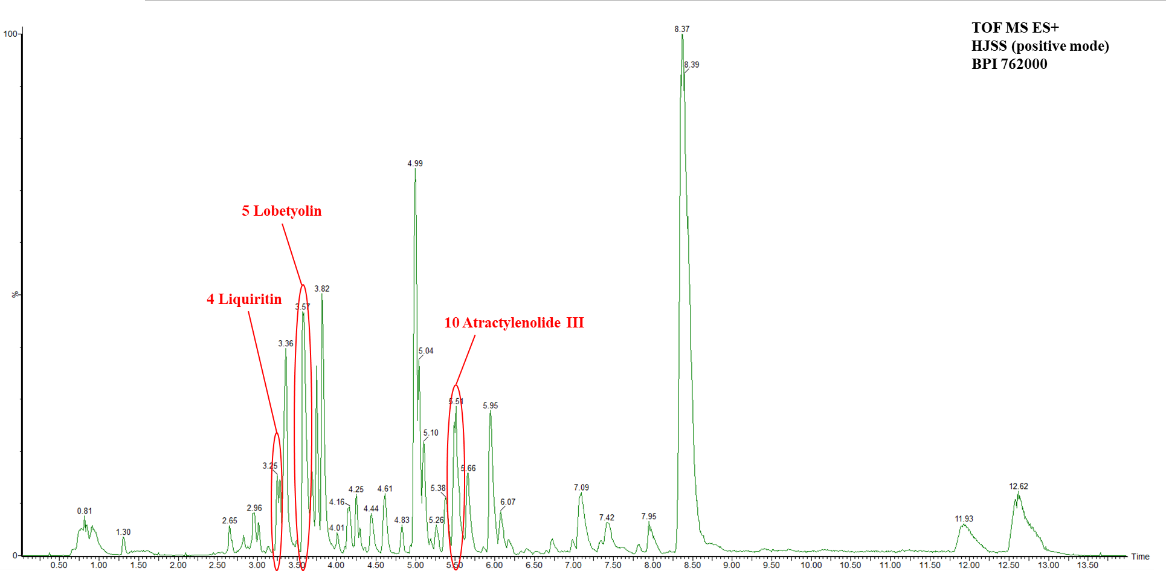


**b**


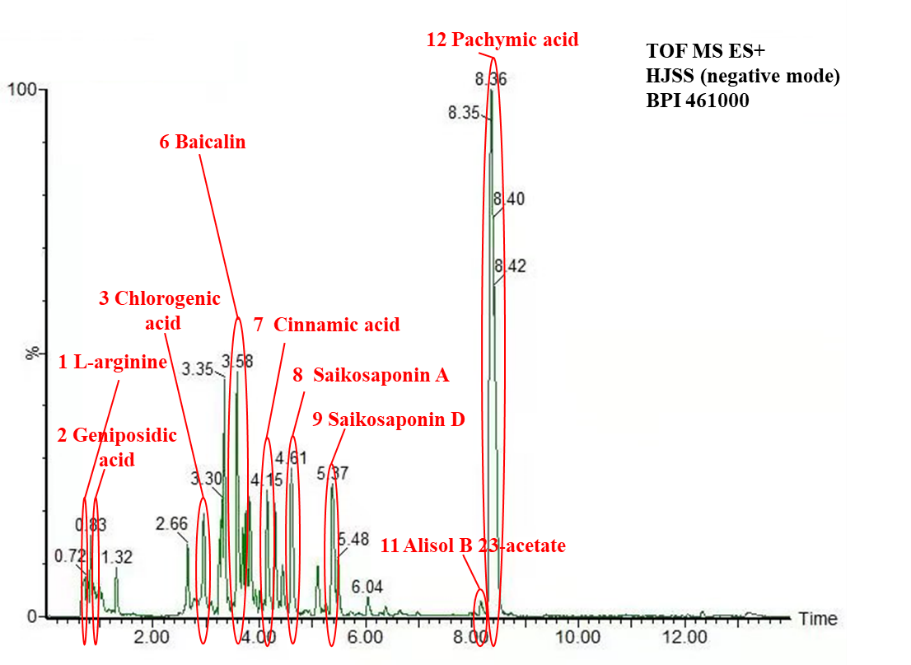


**c**


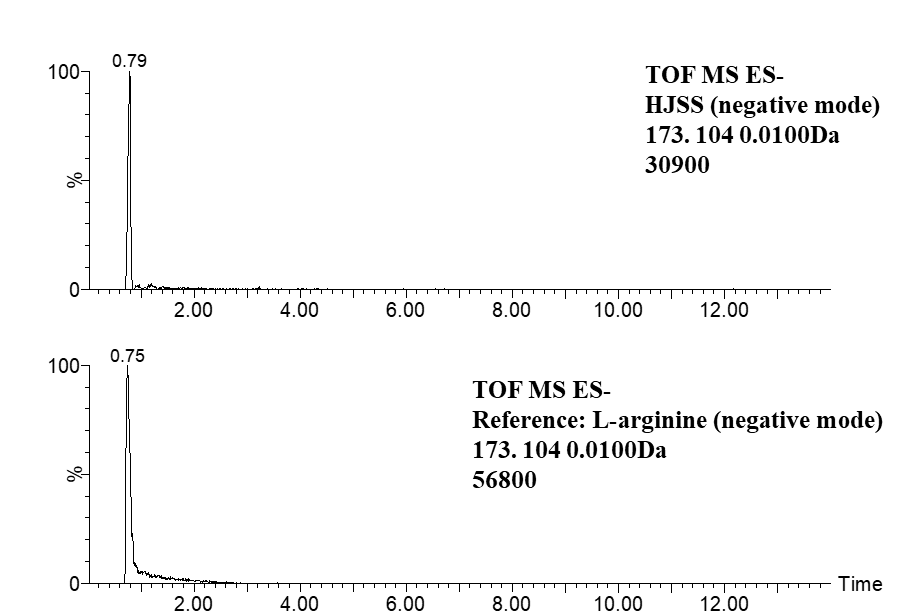


**d**

**
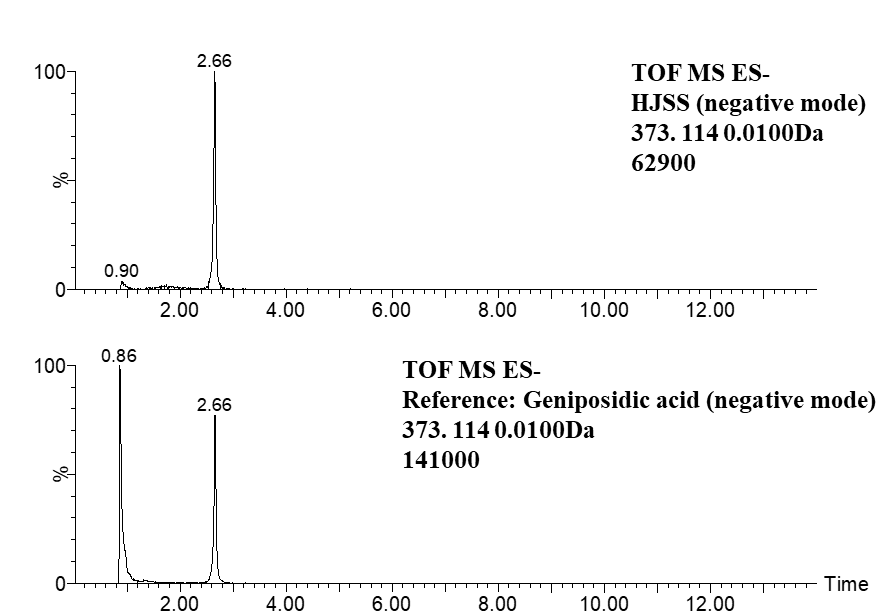
**

**e**

**
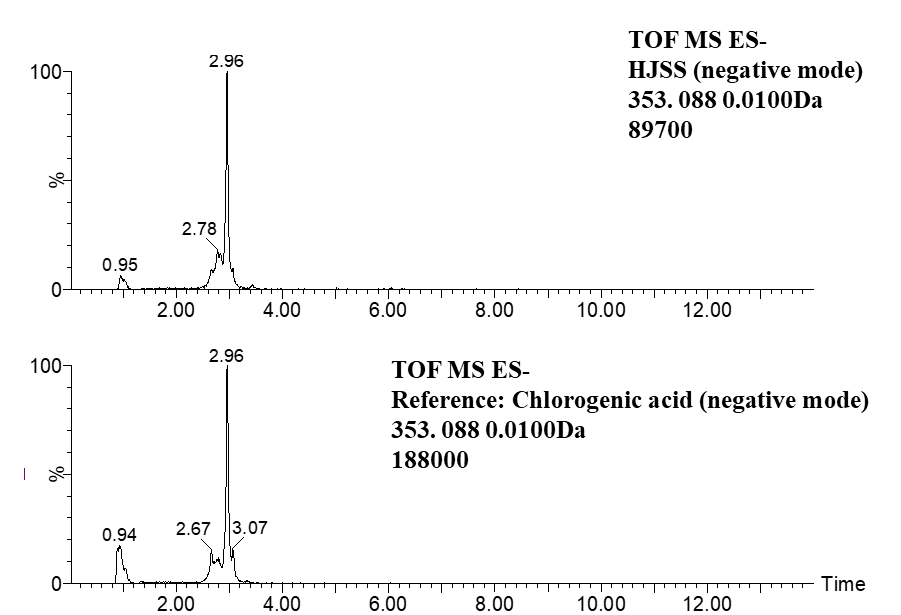
**

**f**

**
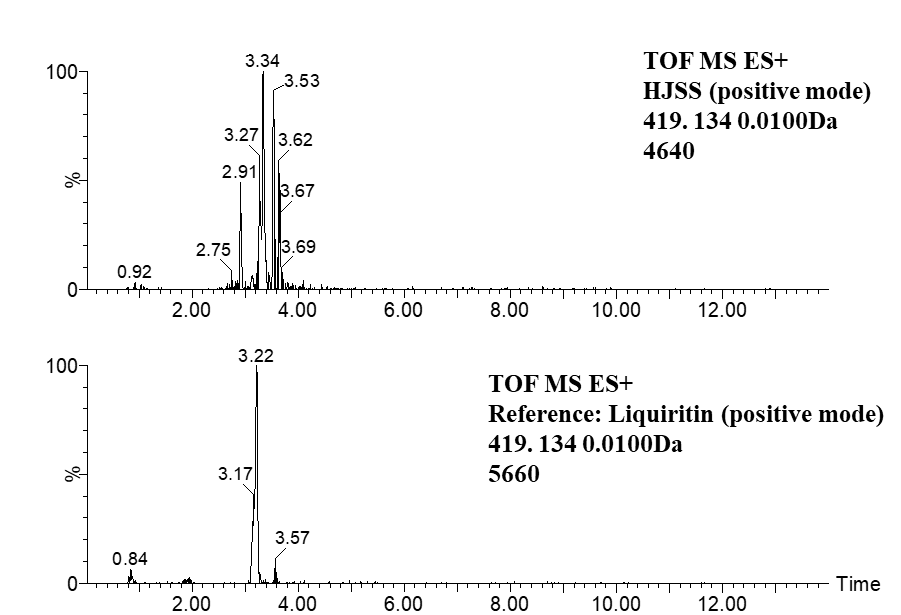
**

**g**

**
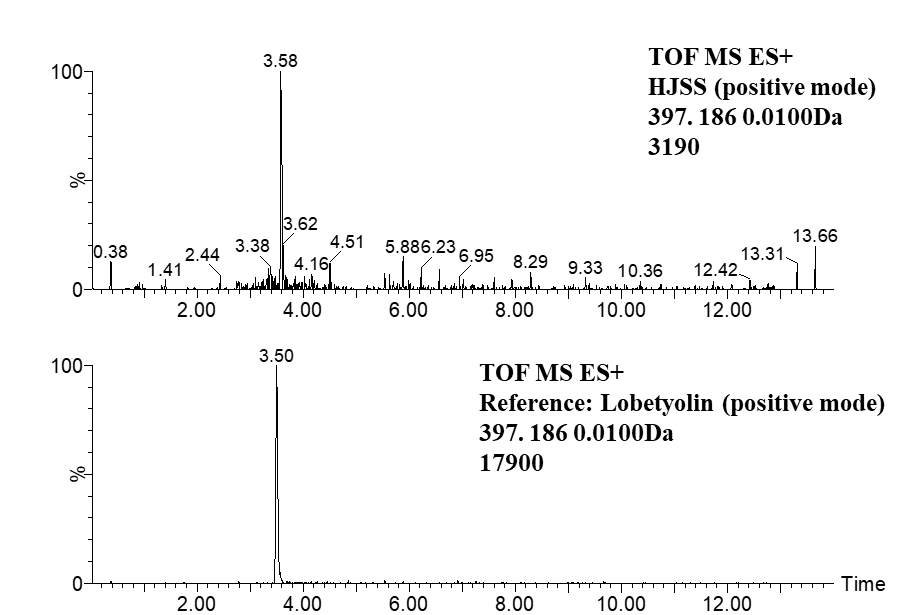
**

**h**

**
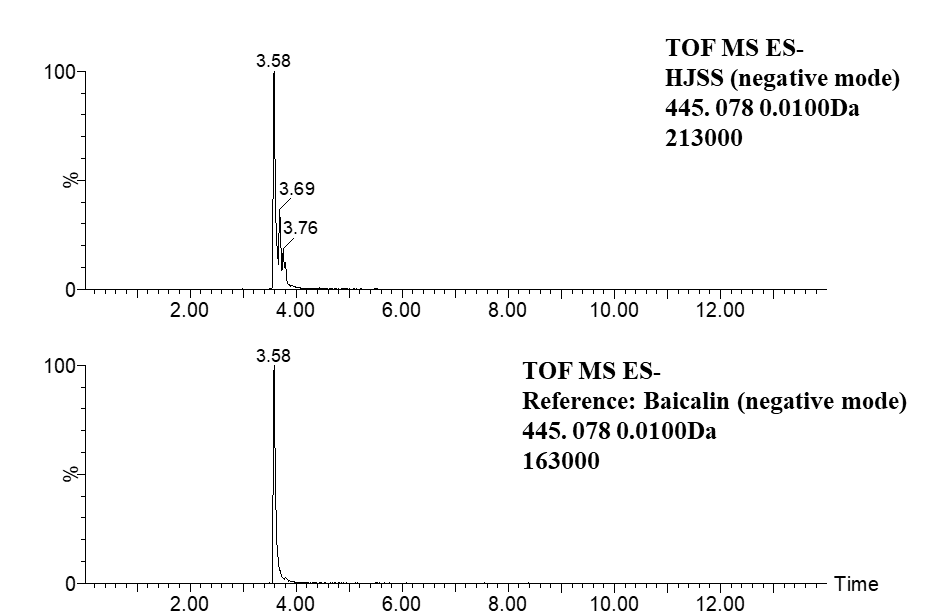
**

**i**

**
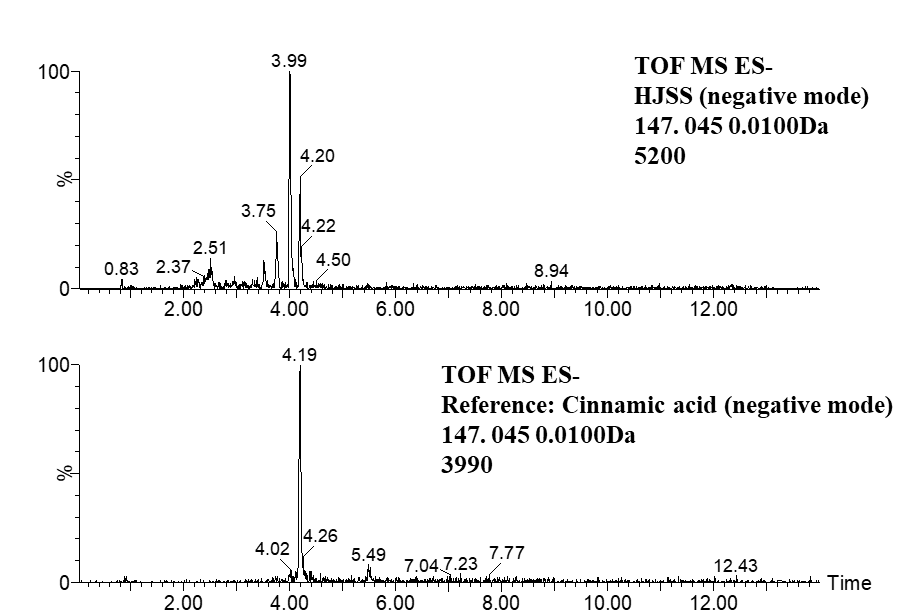
**

**j**

**
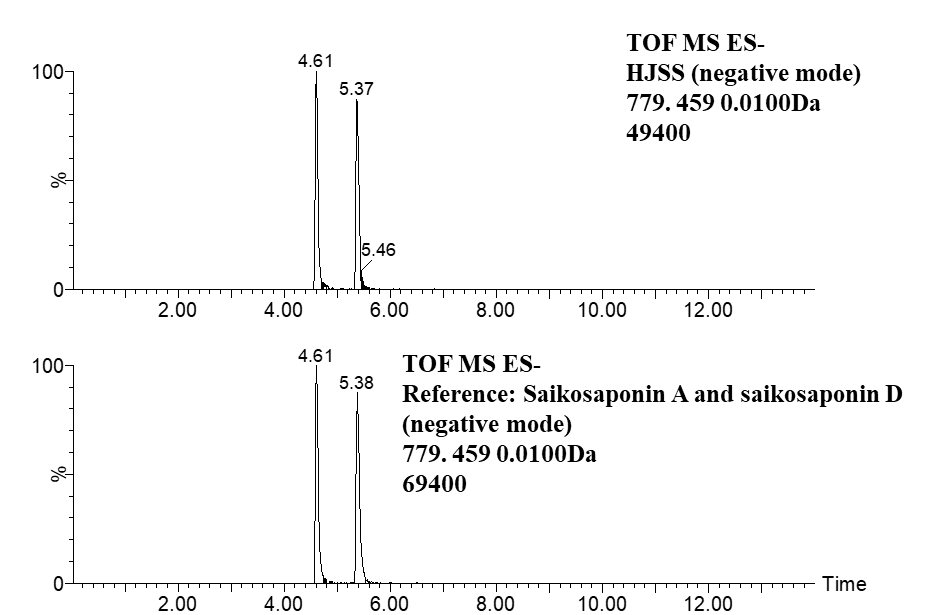
**

**k**

**
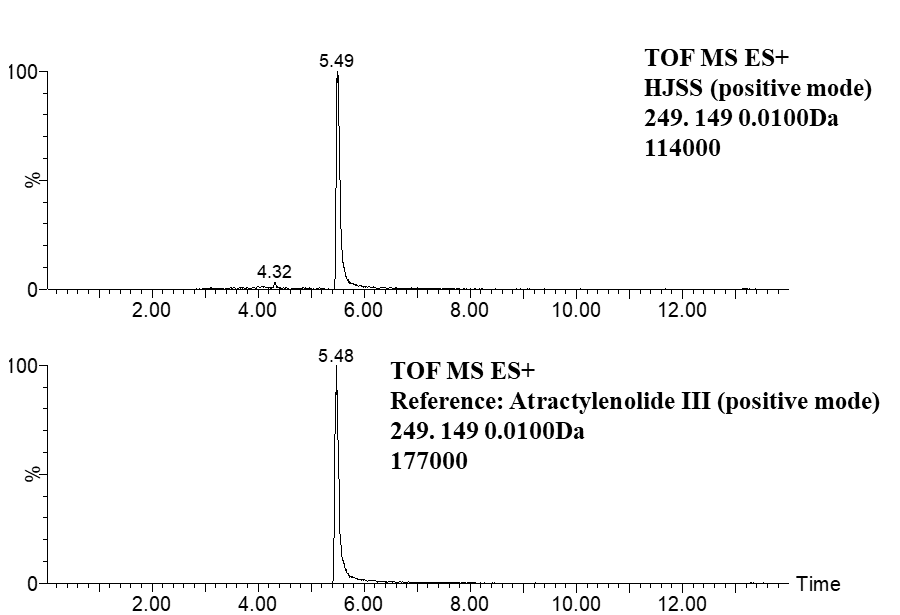
**

**l**

**
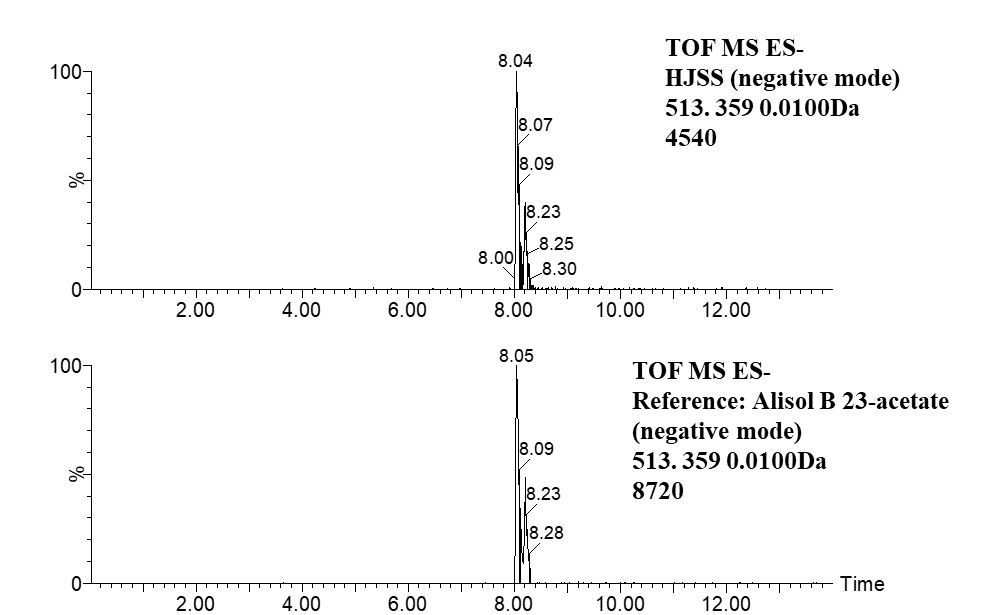
**

**m**

**
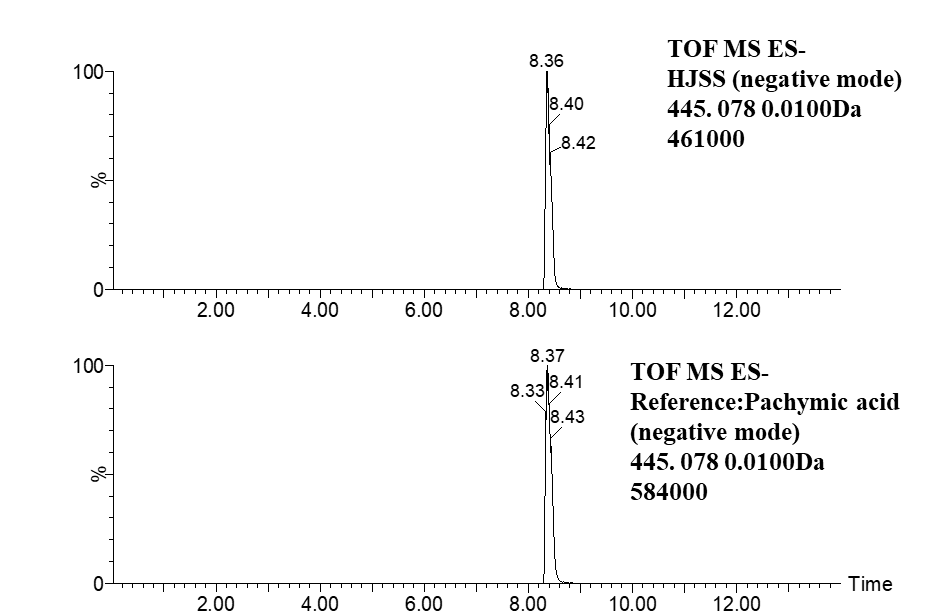
n**

**Figure S2:** The chemical profiles of HJSS using UPLC-MS. (**a, b**) The total ion chromatogram in positive (**a**) and negative ion modes (**b**). (**c-m**) The main bioactive compounds of L-arginine (**c**), geniposidic acid (**d**), chlorogenic acid (**e**), liquiritin (**f**), lobetyolin (**g**), baicalin (**h**), cinnamic acid (**i**), saikosaponin A and saikosaponin D (**j**), atractylenolide III (**k**), alisol B 23-acetate (**l**), pachymic acid (**m**). The bioactive compounds detected in HJSS were confirmed by the reference standards.

**TABLE S1 Detailed** **inclusion and exclusion criterion in our study**

| **Inclusion Criteria** | **Exclusion Criteria** |
| --- | --- |
| **Major Criteria**  1. Age range from 18 to 75.  2. Individuals who test positive for SARS-CoV-2 with RT-PCR. | 1. Pregnant or lactating women. |
|  | 2. Severe primary diseases such as severe combined immune deficiency disease, congenital pulmonary airway malformation, congenital heart disease, and bronchopulmonary dysplasia. |
| **Minor Criteria** | 3. Psychiatric or neurological disorder. |
| 1. Shortness of breath and RR ≥ 30 times/min. | 4. Transfer to other hospitals. |
| 2. SpO_2_ ≤ 93% (at rest). | 5. Other factors such as self-withdrawal during treatment and incomplete clinical data records. |
| 3. PaO_2_/FiO_2_ ≤ 300 mmHg (1mmHg=0.133kPa) on room air at sea level.  *In high-altitude areas (at an altitude of over 1,000 meters above the sea level), PaO_2_/FiO_2_ shall be corrected by the following formula: PaO_2_/FiO_2_ × [Atmospheric pressure (mmHg)/760]. |  |
| 4. Cases with chest imaging that showed obvious lung infiltrates >50% within 24 to 48 h. |  |
| 5. Individuals who have respiratory failure, septic shock, and/or multiple organ failure. |  |

For inclusion criteria, cases should accord with all major criterion and one or more minor criteria. For exclusion criteria, cases were excluded with one or more criterion.

**TABLE S2 The characteristic fragment ions of reference standards in HJSS**

| **Marking**  **peak no.** | **Name** | **RT**  **(min)** | **Ion** | **m/z** |
| --- | --- | --- | --- | --- |
| 1 | L-arginine | 0.75 | [M-H]^-^ | 173.1044 |
| 2 | Geniposidic acid | 0.86 | [M-H]^-^ | 373.1140 |
| 3 | Chlorogenic acid | 2.96 | [M-H]^-^ | 353.0878 |
| 4 | Liquiritin | 3.22 | [M+H]^+^ | 419.1337 |
| 5 | Lobetyolin | 3.57 | [M+H]^+^ | 397.1857 |
| 6 | Baicalin | 3.58 | [M-H]^-^ | 445.0776 |
| 7 | Cinnamic acid | 4.19 | [M-H]^-^ | 147.0452 |
| 8 | Saikosaponin A | 4.61 | [M-H]^-^ | 779.4587 |
| 9 | Saikosaponin D | 5.38 | [M-H]^-^ | 779.4587 |
| 10 | Atractylenolide III | 5.48 | [M+H]^+^ | 249.1485 |
| 11 | Alisol B 23-acetate | 8.05 | [M-H]^-^ | 513.3585 |
| 12 | Pachymic acid | 8.37 | [M-H]^-^ | 527.3742 |

**TABLE S3 Information of active ingredients in HJSS**

| MOL-ID | Molecule name | OB (%) | DL | | Herb |
| --- | --- | --- | --- | --- | --- |
| MOL000098 | quercetin | 46.43335 | 0.27525 | *Pyrrosia lingua* (Thunb.) Farw.,*Plantago asiatica* L.,*Glycyrrhiza uralensis* Fisch.ex DC.,*Bupleurum chinense* DC. | |
| MOL000358 | beta-sitosterol | 36.91391 | 0.75123 | *Pyrrosia lingua* (Thunb.) Farw.,*Neolitsea cassia* (L.) Kosterm.,*Pinellia ternata*(Thunb.) Makino,*Scutellaria baicalensis* Georgi | |
| MOL000422 | kaempferol | 41.88225 | 0.24066 | *Pyrrosia lingua* (Thunb.) Farw.,*Glycyrrhiza uralensis* Fisch.ex DC.,*Bupleurum chinense* DC. | |
| MOL003648 | Inermin | 65.83093 | 0.53754 | *Pyrrosia lingua* (Thunb.) Farw. | |
| MOL003773 | Mangiferolic acid | 36.1593 | 0.84358 | *Pyrrosia lingua* (Thunb.) Farw. | |
| MOL003851 | Isoramanone | 39.96686 | 0.50569 | *Pyrrosia lingua* (Thunb.) Farw. | |
| MOL000359 | sitosterol | 36.91391 | 0.7512 | *Plantago asiatica* L.,*Benincasa hispida*(Thunb.) Cogn.,*Coix lacryma-jobi var. ma-yuen* (Rom.Caill.) Stapf,*Glycyrrhiza uralensis* Fisch.ex DC.,*Neolitsea cassia* (L.) Kosterm.,*Scutellaria baicalensis* Georgi,*Alisma plantago-aquatica subsp. orientale* (Sam.) Sam. | |
| MOL001663 | (4aS,6aR,6aS,6bR,8aR,10R,12aR,14bS)-10-hydroxy-2,2,6a,6b,9,9,12a-heptamethyl-1,3,4,5,6,6a,7,8,8a,10,11,12,13,14b-tetradecahydropicene-4a-carboxylic acid | 32.02801 | 0.75713 | *Plantago asiatica* L. | |
| MOL001735 | Dinatin | 30.97205 | 0.27025 | *Plantago asiatica* L. | |
| MOL005869 | daucostero_qt | 36.91391 | 0.75177 | *Plantago asiatica* L. | |
| MOL007813 | Dihydrotricetin | 58.12059 | 0.27709 | *Plantago asiatica* L. | |
| MOL007819 | Hypolaetin | 33.23916 | 0.27682 | *Plantago asiatica* L. | |
| MOL007835 | orobanchoside_qt | 55.98607 | 0.81893 | *Plantago asiatica* L. | |
| MOL007836 | plantaginin_qt | 54.04431 | 0.24201 | *Plantago asiatica* L. | |
| MOL000449 | Stigmasterol | 43.82985 | 0.75665 | *Coix lacryma-jobi var. ma-yuen* (Rom.Caill.) Stapf,*Benincasa hispida*(Thunb.) Cogn.,*Codonopsis pilosula* (Franch.) Nannf.,*Pinellia ternata*(Thunb.) Makino,*Bupleurum chinense* DC. | |
| MOL000953 | CLR | 37.8739 | 0.67677 | *Coix lacryma-jobi var. ma-yuen* (Rom.Caill.) Stapf | |
| MOL001323 | Sitosterol alpha1 | 43.28127 | 0.78354 | *Coix lacryma-jobi var. ma-yuen* (Rom.Caill.) Stapf | |
| MOL001494 | Mandenol | 41.9962 | 0.19321 | *Coix lacryma-jobi var. ma-yuen* (Rom.Caill.) Stapf | |
| MOL002372 | (6Z,10E,14E,18E)-2,6,10,15,19,23-hexamethyltetracosa-2,6,10,14,18,22-hexaene | 33.54594 | 0.42436 | *Coix lacryma-jobi var. ma-yuen* (Rom.Caill.) Stapf | |
| MOL002882 | [(2R)-2,3-dihydroxypropyl] (Z)-octadec-9-enoate | 34.13108 | 0.29824 | *Coix lacryma-jobi var. ma-yuen* (Rom.Caill.) Stapf | |
| MOL008118 | Coixenolide | 32.39813 | 0.42591 | *Coix lacryma-jobi var. ma-yuen* (Rom.Caill.) Stapf | |
| MOL008121 | 2-Monoolein | 34.23497 | 0.29162 | *Coix lacryma-jobi var. ma-yuen* (Rom.Caill.) Stapf | |
| MOL000279 | Cerevisterol | 37.96383 | 0.77061 | *Poria cocos* (Schw.)Wolf | |
| MOL000282 | ergosta-7,22E-dien-3beta-ol | 43.50709 | 0.71939 | *Poria cocos* (Schw.)Wolf | |
| MOL000073 | ent-Epicatechin | 48.95984 | 0.24162 | *Neolitsea cassia* (L.) Kosterm. | |
| MOL000492 | (+)-catechin | 54.82643 | 0.24164 | *Neolitsea cassia* (L.) Kosterm. | |
| MOL001736 | (-)-taxifolin | 60.50622 | 0.27342 | *Neolitsea cassia* (L.) Kosterm. | |
| MOL000020 | 12-senecioyl-2E,8E,10E-atractylentriol | 62.39647 | 0.22294 | *Atractylodes macrocephala* Koidz. | |
| MOL000021 | 14-acetyl-12-senecioyl-2E,8E,10E-atractylentriol | 60.31287 | 0.30534 | *Atractylodes macrocephala* Koidz. | |
| MOL000022 | 14-acetyl-12-senecioyl-2E,8Z,10E-atractylentriol | 63.37092 | 0.29956 | *Atractylodes macrocephala* Koidz. | |
| MOL000028 | α-Amyrin | 39.51209 | 0.7629 | *Atractylodes macrocephala* Koidz. | |
| MOL000033 | (3S,8S,9S,10R,13R,14S,17R)-10,13-dimethyl-17-[(2R,5S)-5-propan-2-yloctan-2-yl]-2,3,4,7,8,9,11,12,14,15,16,17-dodecahydro-1H-cyclopenta[a]phenanthren-3-ol | 36.22847 | 0.78288 | *Atractylodes macrocephala* Koidz. | |
| MOL000049 | 3β-acetoxyatractylone | 54.06672 | 0.21906 | *Atractylodes macrocephala* Koidz. | |
| MOL000072 | 8β-ethoxy atractylenolide Ⅲ | 35.95092 | 0.21079 | *Atractylodes macrocephala* Koidz. | |
| MOL000830 | Alisol B | 34.47307 | 0.81706 | *Alisma plantago-aquatica subsp. orientale* (Sam.) Sam. | |
| MOL000831 | Alisol B monoacetate | 35.57624 | 0.80629 | *Alisma plantago-aquatica subsp. orientale* (Sam.) Sam. | |
| MOL000832 | alisol,b,23-acetate | 32.51622 | 0.81841 | *Alisma plantago-aquatica subsp. orientale* (Sam.) Sam. | |
| MOL000849 | 16β-methoxyalisol B monoacetate | 32.42724 | 0.7679 | *Alisma plantago-aquatica subsp. orientale* (Sam.) Sam. | |
| MOL000853 | alisol B | 36.76038 | 0.81993 | *Alisma plantago-aquatica subsp. orientale* (Sam.) Sam. | |
| MOL000854 | alisol C | 32.70017 | 0.81507 | *Alisma plantago-aquatica subsp. orientale* (Sam.) Sam. | |
| MOL000856 | alisol C monoacetate | 33.06359 | 0.82763 | *Alisma plantago-aquatica subsp. orientale* (Sam.) Sam. | |
| MOL000862 | [(1S,3R)-1-[(2R)-3,3-dimethyloxiran-2-yl]-3-[(5R,8S,9S,10S,11S,14R)-11-hydroxy-4,4,8,10,14-pentamethyl-3-oxo-1,2,5,6,7,9,11,12,15,16-decahydrocyclopenta[a]phenanthren-17-yl]butyl] acetate | 35.57624 | 0.80765 | *Alisma plantago-aquatica subsp. orientale* (Sam.) Sam. | |
| MOL002464 | 1-Monolinolein | 37.17663 | 0.30249 | *Alisma plantago-aquatica subsp. orientale* (Sam.) Sam. | |
| MOL000273 | (2R)-2-[(3S,5R,10S,13R,14R,16R,17R)-3,16-dihydroxy-4,4,10,13,14-pentamethyl-2,3,5,6,12,15,16,17-octahydro-1H-cyclopenta[a]phenanthren-17-yl]-6-methylhept-5-enoic acid | 30.93214 | 0.81281 | *Poria cocos* (Schw.)Wolf | |
| MOL000275 | trametenolic acid | 38.7115 | 0.80199 | *Poria cocos* (Schw.)Wolf | |
| MOL000276 | 7,9(11)-dehydropachymic acid | 35.10589 | 0.81091 | *Poria cocos* (Schw.)Wolf | |
| MOL000280 | (2R)-2-[(3S,5R,10S,13R,14R,16R,17R)-3,16-dihydroxy-4,4,10,13,14-pentamethyl-2,3,5,6,12,15,16,17-octahydro-1H-cyclopenta[a]phenanthren-17-yl]-5-isopropyl-hex-5-enoic acid | 31.07206 | 0.81528 | *Poria cocos* (Schw.)Wolf | |
| MOL000283 | Ergosterol peroxide | 40.36268 | 0.81255 | *Poria cocos* (Schw.)Wolf | |
| MOL000285 | (2R)-2-[(5R,10S,13R,14R,16R,17R)-16-hydroxy-3-keto-4,4,10,13,14-pentamethyl-1,2,5,6,12,15,16,17-octahydrocyclopenta[a]phenanthren-17-yl]-5-isopropyl-hex-5-enoic acid | 38.25516 | 0.82014 | *Poria cocos* (Schw.)Wolf | |
| MOL000287 | 3beta-Hydroxy-24-methylene-8-lanostene-21-oic acid | 38.69991 | 0.8095 | *Poria cocos* (Schw.)Wolf | |
| MOL000289 | pachymic acid | 33.62792 | 0.81076 | *Poria cocos* (Schw.)Wolf | |
| MOL000290 | Poricoic acid A | 30.60695 | 0.76152 | *Poria cocos* (Schw.)Wolf | |
| MOL000291 | Poricoic acid B | 30.5246 | 0.7463 | *Poria cocos* (Schw.)Wolf | |
| MOL000292 | poricoic acid C | 38.15136 | 0.74643 | *Poria cocos* (Schw.)Wolf | |
| MOL000296 | hederagenin | 36.91391 | 0.75072 | *Poria cocos* (Schw.)Wolf | |
| MOL000300 | dehydroeburicoic acid | 44.1723 | 0.83458 | *Poria cocos* (Schw.)Wolf | |
| MOL000006 | luteolin | 36.16263 | 0.24552 | *Codonopsis pilosula* (Franch.) Nannf. | |
| MOL001006 | poriferasta-7,22E-dien-3beta-ol | 42.97937 | 0.75555 | *Codonopsis pilosula* (Franch.) Nannf. | |
| MOL002140 | Perlolyrine | 65.94775 | 0.2747 | *Codonopsis pilosula* (Franch.) Nannf. | |
| MOL002879 | Diop | 43.59333 | 0.39247 | *Codonopsis pilosula* (Franch.) Nannf. | |
| MOL003036 | ZINC03978781 | 43.82985 | 0.75647 | *Codonopsis pilosula* (Franch.) Nannf. | |
| MOL003896 | 7-Methoxy-2-methyl isoflavone | 42.56474 | 0.19946 | *Codonopsis pilosula* (Franch.) Nannf.,*Glycyrrhiza uralensis* Fisch.ex DC. | |
| MOL004355 | Spinasterol | 42.97937 | 0.75534 | *Codonopsis pilosula* (Franch.) Nannf. | |
| MOL004492 | Chrysanthemaxanthin | 38.72398 | 0.58352 | *Codonopsis pilosula* (Franch.) Nannf. | |
| MOL005321 | Frutinone A | 65.90373 | 0.34184 | *Codonopsis pilosula* (Franch.) Nannf. | |
| MOL006554 | Taraxerol | 38.40254 | 0.76677 | *Codonopsis pilosula* (Franch.) Nannf. | |
| MOL006774 | stigmast-7-enol | 37.42312 | 0.75133 | *Codonopsis pilosula* (Franch.) Nannf. | |
| MOL007059 | 3-beta-Hydroxymethyllenetanshiquinone | 32.16103 | 0.40894 | *Codonopsis pilosula* (Franch.) Nannf. | |
| MOL007514 | methyl icosa-11,14-dienoate | 39.66706 | 0.22908 | *Codonopsis pilosula* (Franch.) Nannf. | |
| MOL008391 | 5alpha-Stigmastan-3,6-dione | 33.1154 | 0.79021 | *Codonopsis pilosula* (Franch.) Nannf. | |
| MOL008393 | 7-(beta-Xylosyl)cephalomannine_qt | 38.32746 | 0.28646 | *Codonopsis pilosula* (Franch.) Nannf. | |
| MOL008397 | Daturilin | 50.36513 | 0.76801 | *Codonopsis pilosula* (Franch.) Nannf. | |
| MOL008400 | glycitein | 50.47891 | 0.23826 | *Codonopsis pilosula* (Franch.) Nannf. | |
| MOL008406 | Spinoside A | 39.96686 | 0.40288 | *Codonopsis pilosula* (Franch.) Nannf. | |
| MOL008407 | (8S,9S,10R,13R,14S,17R)-17-[(E,2R,5S)-5-ethyl-6-methylhept-3-en-2-yl]-10,13-dimethyl-1,2,4,7,8,9,11,12,14,15,16,17-dodecahydrocyclopenta[a]phenanthren-3-one | 45.40462 | 0.76174 | *Codonopsis pilosula* (Franch.) Nannf. | |
| MOL008411 | 11-Hydroxyrankinidine | 40.00276 | 0.66203 | *Codonopsis pilosula* (Franch.) Nannf. | |
| MOL000211 | Mairin | 55.37707 | 0.7761 | *Glycyrrhiza uralensis* Fisch.ex DC. | |
| MOL000239 | Jaranol | 50.82882 | 0.29148 | *Glycyrrhiza uralensis* Fisch.ex DC. | |
| MOL000354 | isorhamnetin | 49.60438 | 0.306 | *Glycyrrhiza uralensis* Fisch.ex DC.,*Bupleurum chinense* DC. | |
| MOL000392 | formononetin | 69.67388 | 0.21202 | *Glycyrrhiza uralensis* Fisch.ex DC. | |
| MOL000417 | Calycosin | 47.75183 | 0.24278 | *Glycyrrhiza uralensis* Fisch.ex DC. | |
| MOL000497 | licochalcone a | 40.78965 | 0.28517 | *Glycyrrhiza uralensis* Fisch.ex DC. | |
| MOL000500 | Vestitol | 74.65519 | 0.20935 | *Glycyrrhiza uralensis* Fisch.ex DC. | |
| MOL001484 | Inermine | 75.18306 | 0.53754 | *Glycyrrhiza uralensis* Fisch.ex DC. | |
| MOL001792 | DFV | 32.76272 | 0.18316 | *Glycyrrhiza uralensis* Fisch.ex DC. | |
| MOL002311 | Glycyrol | 90.77578 | 0.66819 | *Glycyrrhiza uralensis* Fisch.ex DC. | |
| MOL002565 | Medicarpin | 49.21982 | 0.3351 | *Glycyrrhiza uralensis* Fisch.ex DC. | |
| MOL003656 | Lupiwighteone | 51.63569 | 0.36739 | *Glycyrrhiza uralensis* Fisch.ex DC. | |
| MOL004328 | naringenin | 59.2939 | 0.21128 | *Glycyrrhiza uralensis* Fisch.ex DC. | |
| MOL004805 | (2S)-2-[4-hydroxy-3-(3-methylbut-2-enyl)phenyl]-8,8-dimethyl-2,3-dihydropyrano[2,3-f]chromen-4-one | 31.78703 | 0.72403 | *Glycyrrhiza uralensis* Fisch.ex DC. | |
| MOL004806 | euchrenone | 30.28726 | 0.57386 | *Glycyrrhiza uralensis* Fisch.ex DC. | |
| MOL004808 | glyasperin B | 65.22439 | 0.43851 | *Glycyrrhiza uralensis* Fisch.ex DC. | |
| MOL004810 | glyasperin F | 75.8368 | 0.53514 | *Glycyrrhiza uralensis* Fisch.ex DC. | |
| MOL004811 | Glyasperin C | 45.56381 | 0.39947 | *Glycyrrhiza uralensis* Fisch.ex DC. | |
| MOL004814 | Isotrifoliol | 31.94479 | 0.42422 | *Glycyrrhiza uralensis* Fisch.ex DC. | |
| MOL004815 | (E)-1-(2,4-dihydroxyphenyl)-3-(2,2-dimethylchromen-6-yl)prop-2-en-1-one | 39.61686 | 0.35077 | *Glycyrrhiza uralensis* Fisch.ex DC. | |
| MOL004820 | kanzonols W | 50.48008 | 0.51704 | *Glycyrrhiza uralensis* Fisch.ex DC. | |
| MOL004824 | (2S)-6-(2,4-dihydroxyphenyl)-2-(2-hydroxypropan-2-yl)-4-methoxy-2,3-dihydrofuro[3,2-g]chromen-7-one | 60.25041 | 0.63433 | *Glycyrrhiza uralensis* Fisch.ex DC. | |
| MOL004827 | Semilicoisoflavone B | 48.77755 | 0.54732 | *Glycyrrhiza uralensis* Fisch.ex DC. | |
| MOL004828 | Glepidotin A | 44.72187 | 0.34685 | *Glycyrrhiza uralensis* Fisch.ex DC. | |
| MOL004829 | Glepidotin B | 64.46292 | 0.34485 | *Glycyrrhiza uralensis* Fisch.ex DC. | |
| MOL004833 | Phaseolinisoflavan | 32.00811 | 0.44538 | *Glycyrrhiza uralensis* Fisch.ex DC. | |
| MOL004835 | Glypallichalcone | 61.59706 | 0.18993 | *Glycyrrhiza uralensis* Fisch.ex DC. | |
| MOL004838 | 8-(6-hydroxy-2-benzofuranyl)-2,2-dimethyl-5-chromenol | 58.43728 | 0.38106 | *Glycyrrhiza uralensis* Fisch.ex DC. | |
| MOL004841 | Licochalcone B | 76.75735 | 0.1935 | *Glycyrrhiza uralensis* Fisch.ex DC. | |
| MOL004848 | licochalcone G | 49.25496 | 0.32325 | *Glycyrrhiza uralensis* Fisch.ex DC. | |
| MOL004849 | 3-(2,4-dihydroxyphenyl)-8-(1,1-dimethylprop-2-enyl)-7-hydroxy-5-methoxy-coumarin | 59.62247 | 0.42894 | *Glycyrrhiza uralensis* Fisch.ex DC. | |
| MOL004855 | Licoricone | 63.57846 | 0.4712 | *Glycyrrhiza uralensis* Fisch.ex DC. | |
| MOL004856 | Gancaonin A | 51.07519 | 0.40378 | *Glycyrrhiza uralensis* Fisch.ex DC. | |
| MOL004857 | Gancaonin B | 48.7944 | 0.44924 | *Glycyrrhiza uralensis* Fisch.ex DC. | |
| MOL004860 | licorice glycoside E | 32.88743 | 0.27218 | *Glycyrrhiza uralensis* Fisch.ex DC. | |
| MOL004863 | 3-(3,4-dihydroxyphenyl)-5,7-dihydroxy-8-(3-methylbut-2-enyl)chromone | 66.37125 | 0.41392 | *Glycyrrhiza uralensis* Fisch.ex DC. | |
| MOL004864 | 5,7-dihydroxy-3-(4-methoxyphenyl)-8-(3-methylbut-2-enyl)chromone | 30.48878 | 0.41002 | *Glycyrrhiza uralensis* Fisch.ex DC. | |
| MOL004866 | 2-(3,4-dihydroxyphenyl)-5,7-dihydroxy-6-(3-methylbut-2-enyl)chromone | 44.15196 | 0.41482 | *Glycyrrhiza uralensis* Fisch.ex DC. | |
| MOL004879 | Glycyrin | 52.60657 | 0.47466 | *Glycyrrhiza uralensis* Fisch.ex DC. | |
| MOL004882 | Licocoumarone | 33.21085 | 0.3568 | *Glycyrrhiza uralensis* Fisch.ex DC. | |
| MOL004883 | Licoisoflavone | 41.61022 | 0.41646 | *Glycyrrhiza uralensis* Fisch.ex DC. | |
| MOL004884 | Licoisoflavone B | 38.92871 | 0.54714 | *Glycyrrhiza uralensis* Fisch.ex DC. | |
| MOL004885 | licoisoflavanone | 52.46625 | 0.54488 | *Glycyrrhiza uralensis* Fisch.ex DC. | |
| MOL004891 | shinpterocarpin | 80.29528 | 0.72746 | *Glycyrrhiza uralensis* Fisch.ex DC. | |
| MOL004898 | (E)-3-[3,4-dihydroxy-5-(3-methylbut-2-enyl)phenyl]-1-(2,4-dihydroxyphenyl)prop-2-en-1-one | 46.26792 | 0.3062 | *Glycyrrhiza uralensis* Fisch.ex DC. | |
| MOL004903 | liquiritin | 65.69011 | 0.73893 | *Glycyrrhiza uralensis* Fisch.ex DC. | |
| MOL004904 | licopyranocoumarin | 80.36001 | 0.6535 | *Glycyrrhiza uralensis* Fisch.ex DC. | |
| MOL004905 | 3,22-Dihydroxy-11-oxo-delta(12)-oleanene-27-alpha-methoxycarbonyl-29-oic acid | 34.31942 | 0.54718 | *Glycyrrhiza uralensis* Fisch.ex DC. | |
| MOL004907 | Glyzaglabrin | 61.06889 | 0.35347 | *Glycyrrhiza uralensis* Fisch.ex DC. | |
| MOL004908 | Glabridin | 53.24514 | 0.46967 | *Glycyrrhiza uralensis* Fisch.ex DC. | |
| MOL004910 | Glabranin | 52.89566 | 0.31208 | *Glycyrrhiza uralensis* Fisch.ex DC. | |
| MOL004911 | Glabrene | 46.26686 | 0.43902 | *Glycyrrhiza uralensis* Fisch.ex DC. | |
| MOL004912 | Glabrone | 52.51217 | 0.49645 | *Glycyrrhiza uralensis* Fisch.ex DC. | |
| MOL004913 | 1,3-dihydroxy-9-methoxy-6-benzofurano[3,2-c]chromenone | 48.14154 | 0.42831 | *Glycyrrhiza uralensis* Fisch.ex DC. | |
| MOL004914 | 1,3-dihydroxy-8,9-dimethoxy-6-benzofurano[3,2-c]chromenone | 62.90135 | 0.52759 | *Glycyrrhiza uralensis* Fisch.ex DC. | |
| MOL004915 | Eurycarpin A | 43.27728 | 0.37429 | *Glycyrrhiza uralensis* Fisch.ex DC. | |
| MOL004917 | glycyroside | 37.25032 | 0.79156 | *Glycyrrhiza uralensis* Fisch.ex DC. | |
| MOL004924 | (-)-Medicocarpin | 40.99397 | 0.95059 | *Glycyrrhiza uralensis* Fisch.ex DC. | |
| MOL004935 | Sigmoidin-B | 34.88109 | 0.41455 | *Glycyrrhiza uralensis* Fisch.ex DC. | |
| MOL004941 | (2R)-7-hydroxy-2-(4-hydroxyphenyl)chroman-4-one | 71.12299 | 0.18303 | *Glycyrrhiza uralensis* Fisch.ex DC. | |
| MOL004945 | (2S)-7-hydroxy-2-(4-hydroxyphenyl)-8-(3-methylbut-2-enyl)chroman-4-one | 36.56537 | 0.32291 | *Glycyrrhiza uralensis* Fisch.ex DC. | |
| MOL004948 | Isoglycyrol | 44.69923 | 0.83845 | *Glycyrrhiza uralensis* Fisch.ex DC. | |
| MOL004949 | Isolicoflavonol | 45.16999 | 0.41859 | *Glycyrrhiza uralensis* Fisch.ex DC. | |
| MOL004957 | HMO | 38.36542 | 0.21067 | *Glycyrrhiza uralensis* Fisch.ex DC. | |
| MOL004959 | 1-Methoxyphaseollidin | 69.98098 | 0.63739 | *Glycyrrhiza uralensis* Fisch.ex DC. | |
| MOL004961 | Quercetin der. | 46.44939 | 0.3343 | *Glycyrrhiza uralensis* Fisch.ex DC. | |
| MOL004966 | 3'-Hydroxy-4'-O-Methylglabridin | 43.71495 | 0.57406 | *Glycyrrhiza uralensis* Fisch.ex DC. | |
| MOL004974 | 3'-Methoxyglabridin | 46.16151 | 0.57393 | *Glycyrrhiza uralensis* Fisch.ex DC. | |
| MOL004978 | 2-[(3R)-8,8-dimethyl-3,4-dihydro-2H-pyrano[6,5-f]chromen-3-yl]-5-methoxyphenol | 36.21429 | 0.52122 | *Glycyrrhiza uralensis* Fisch.ex DC. | |
| MOL004980 | Inflacoumarin A | 39.7091 | 0.32613 | *Glycyrrhiza uralensis* Fisch.ex DC. | |
| MOL004985 | icos-5-enoic acid | 30.70294 | 0.19725 | *Glycyrrhiza uralensis* Fisch.ex DC. | |
| MOL004988 | Kanzonol F | 32.46833 | 0.89364 | *Glycyrrhiza uralensis* Fisch.ex DC. | |
| MOL004989 | 6-prenylated eriodictyol | 39.22383 | 0.41259 | *Glycyrrhiza uralensis* Fisch.ex DC. | |
| MOL004990 | 7,2',4'-trihydroxy－5-methoxy-3－arylcoumarin | 83.71437 | 0.27136 | *Glycyrrhiza uralensis* Fisch.ex DC. | |
| MOL004991 | 7-Acetoxy-2-methylisoflavone | 38.92333 | 0.26217 | *Glycyrrhiza uralensis* Fisch.ex DC. | |
| MOL004993 | 8-prenylated eriodictyol | 53.79476 | 0.40383 | *Glycyrrhiza uralensis* Fisch.ex DC. | |
| MOL004996 | gadelaidic acid | 30.70294 | 0.19725 | *Glycyrrhiza uralensis* Fisch.ex DC. | |
| MOL005000 | Gancaonin G | 60.43521 | 0.39404 | *Glycyrrhiza uralensis* Fisch.ex DC. | |
| MOL005001 | Gancaonin H | 50.10372 | 0.78416 | *Glycyrrhiza uralensis* Fisch.ex DC. | |
| MOL005003 | Licoagrocarpin | 58.8139 | 0.58498 | *Glycyrrhiza uralensis* Fisch.ex DC. | |
| MOL005007 | Glyasperins M | 72.67081 | 0.59274 | *Glycyrrhiza uralensis* Fisch.ex DC. | |
| MOL005008 | Glycyrrhiza flavonol A | 41.27528 | 0.59512 | *Glycyrrhiza uralensis* Fisch.ex DC. | |
| MOL005012 | Licoagroisoflavone | 57.28224 | 0.48679 | *Glycyrrhiza uralensis* Fisch.ex DC. | |
| MOL005013 | 18α-hydroxyglycyrrhetic acid | 41.16139 | 0.7091 | *Glycyrrhiza uralensis* Fisch.ex DC. | |
| MOL005016 | Odoratin | 49.94822 | 0.30487 | *Glycyrrhiza uralensis* Fisch.ex DC. | |
| MOL005017 | Phaseol | 78.76622 | 0.57867 | *Glycyrrhiza uralensis* Fisch.ex DC. | |
| MOL005018 | Xambioona | 54.84916 | 0.87419 | *Glycyrrhiza uralensis* Fisch.ex DC. | |
| MOL005020 | dehydroglyasperins C | 53.82326 | 0.37006 | *Glycyrrhiza uralensis* Fisch.ex DC. | |
| MOL000519 | coniferin | 31.11 | 0.32308 | *Pinellia ternata*(Thunb.) Makino | |
| MOL001755 | 24-Ethylcholest-4-en-3-one | 36.08361 | 0.75703 | *Pinellia ternata*(Thunb.) Makino | |
| MOL002670 | Cavidine | 35.64183 | 0.80513 | *Pinellia ternata*(Thunb.) Makino | |
| MOL002714 | baicalein | 33.51892 | 0.20888 | *Pinellia ternata*(Thunb.) Makino,*Scutellaria baicalensis* Georgi | |
| MOL002776 | Baicalin | 40.12361 | 0.75264 | *Pinellia ternata*(Thunb.) Makino,*Bupleurum chinense* DC. | |
| MOL003578 | Cycloartenol | 38.68566 | 0.78093 | *Pinellia ternata*(Thunb.) Makino | |
| MOL005030 | gondoic acid | 30.70294 | 0.19743 | *Pinellia ternata*(Thunb.) Makino | |
| MOL006936 | 10,13-eicosadienoic | 39.99355 | 0.20012 | *Pinellia ternata*(Thunb.) Makino | |
| MOL006937 | 12,13-epoxy-9-hydroxynonadeca-7,10-dienoic acid | 42.15218 | 0.24248 | *Pinellia ternata*(Thunb.) Makino | |
| MOL006957 | (3S,6S)-3-(benzyl)-6-(4-hydroxybenzyl)piperazine-2,5-quinone | 46.8889 | 0.26989 | *Pinellia ternata*(Thunb.) Makino | |
| MOL006967 | beta-D-Ribofuranoside, xanthine-9 | 44.71879 | 0.20816 | *Pinellia ternata*(Thunb.) Makino | |
| MOL000173 | wogonin | 30.68457 | 0.22942 | *Scutellaria baicalensis* Georgi | |
| MOL000228 | (2R)-7-hydroxy-5-methoxy-2-phenylchroman-4-one | 55.23317 | 0.20163 | *Scutellaria baicalensis* Georgi | |
| MOL000525 | Norwogonin | 39.40397 | 0.20723 | *Scutellaria baicalensis* Georgi | |
| MOL000552 | 5,2'-Dihydroxy-6,7,8-trimethoxyflavone | 31.71246 | 0.35462 | *Scutellaria baicalensis* Georgi | |
| MOL001689 | acacetin | 34.97357 | 0.24082 | *Scutellaria baicalensis* Georgi | |
| MOL002908 | 5,8,2'-Trihydroxy-7-methoxyflavone | 37.00837 | 0.26546 | *Scutellaria baicalensis* Georgi | |
| MOL002909 | 5,7,2,5-tetrahydroxy-8,6-dimethoxyflavone | 33.81583 | 0.44739 | *Scutellaria baicalensis* Georgi | |
| MOL002910 | Carthamidin | 41.15096 | 0.24189 | *Scutellaria baicalensis* Georgi | |
| MOL002911 | 2,6,2',4'-tetrahydroxy-6'-methoxychaleone | 69.03988 | 0.21994 | *Scutellaria baicalensis* Georgi | |
| MOL002913 | Dihydrobaicalin_qt | 40.03778 | 0.20722 | *Scutellaria baicalensis* Georgi | |
| MOL002914 | Eriodyctiol (flavanone) | 41.35043 | 0.2436 | *Scutellaria baicalensis* Georgi | |
| MOL002915 | Salvigenin | 49.06593 | 0.33279 | *Scutellaria baicalensis* Georgi | |
| MOL002917 | 5,2',6'-Trihydroxy-7,8-dimethoxyflavone | 45.04743 | 0.33057 | *Scutellaria baicalensis* Georgi | |
| MOL002925 | 5,7,2',6'-Tetrahydroxyflavone | 37.01349 | 0.24382 | *Scutellaria baicalensis* Georgi | |
| MOL002926 | dihydrooroxylin A | 38.71507 | 0.22987 | *Scutellaria baicalensis* Georgi | |
| MOL002927 | Skullcapflavone II | 69.51043 | 0.4379 | *Scutellaria baicalensis* Georgi | |
| MOL002928 | oroxylin a | 41.36757 | 0.23233 | *Scutellaria baicalensis* Georgi | |
| MOL002932 | Panicolin | 76.25705 | 0.2915 | *Scutellaria baicalensis* Georgi | |
| MOL002933 | 5,7,4'-Trihydroxy-8-methoxyflavone | 36.562 | 0.26666 | *Scutellaria baicalensis* Georgi | |
| MOL002934 | NEOBAICALEIN | 104.3446 | 0.43917 | *Scutellaria baicalensis* Georgi | |
| MOL002937 | DIHYDROOROXYLIN | 66.06174 | 0.23057 | *Scutellaria baicalensis* Georgi | |
| MOL000490 | petunidin | 30.04554 | 0.30712 | *Bupleurum chinense* DC. | |
| MOL001645 | Linoleyl acetate | 42.10077 | 0.19845 | *Bupleurum chinense* DC. | |
| MOL004598 | 3,5,6,7-tetramethoxy-2-(3,4,5-trimethoxyphenyl)chromone | 31.97496 | 0.59317 | *Bupleurum chinense* DC. | |
| MOL004609 | Areapillin | 48.96435 | 0.41394 | *Bupleurum chinense* DC. | |
| MOL004624 | Longikaurin A | 47.72215 | 0.53015 | *Bupleurum chinense* DC. | |
| MOL004628 | Octalupine | 47.82225 | 0.27864 | *Bupleurum chinense* DC. | |
| MOL004644 | Sainfuran | 79.90979 | 0.23331 | *Bupleurum chinense* DC. | |
| MOL004648 | Troxerutin | 31.59657 | 0.28256 | *Bupleurum chinense* DC. | |
| MOL004653 | (+)-Anomalin | 46.05534 | 0.6566 | *Bupleurum chinense* DC. | |
| MOL004702 | saikosaponin c_qt | 30.50493 | 0.63193 | *Bupleurum chinense* DC. | |
| MOL004718 | α-spinasterol | 42.97937 | 0.75693 | *Bupleurum chinense* DC. | |
| MOL013187 | Cubebin | 57.12813 | 0.63988 | *Bupleurum chinense* DC. | |
